# Unraveling the Role of Autotaxin and Lysophosphatidic Acid in Alzheimer’s Disease: From Molecular Mechanisms to Therapeutic Potential

**DOI:** 10.3390/ijms26157068

**Published:** 2025-07-23

**Authors:** Jesús García-de Soto, Mónica Castro-Mosquera, Jessica María Pouso-Diz, Alejandro Fernández-Cabrera, Mariña Rodríguez-Arrizabalaga, Manuel Debasa-Mouce, Javier Camino-Castiñeiras, Anxo Manuel Minguillón Pereiro, Marta Aramburu-Núñez, Daniel Romaus-Sanjurjo, José Manuel Aldrey, Robustiano Pego-Reigosa, Juan Manuel Pías-Peleteiro, Tomás Sobrino, Alberto Ouro

**Affiliations:** 1 NeuroAging Group (NEURAL), Clinical Neurosciences Research Laboratory (LINC), Health Research Institute of Santiago de Compostela (IDIS), 15706 Santiago de Compostela, Spain; jesus.garcia.de.soto@sergas.es (J.G.-d.S.); monicacastro.mosquera@usc.es (M.C.-M.); jessica.maria.pouso.diz@sergas.es (J.M.P.-D.); marina.rodriguez.arrizabalaga@sergas.es (M.R.-A.); manuel.debasa@rai.usc.gal (M.D.-M.); antoniojavier.camino.castineiras@sergas.es (J.C.-C.); anxo.manuel.minguillon.pereiro@sergas.es (A.M.M.P.); marta.aramburu.nunez@sergas.es (M.A.-N.); daniel.romaus.sanjurjo@sergas.es (D.R.-S.); jose.manuel.aldrey.vazquez@sergas.es (J.M.A.); juan.manuel.pias.peleteiro@sergas.es (J.M.P.-P.); 2 Servicio de Neurología, Hospital Universitario Lucus Augusti, 27003 Lugo, Spain; alejandrofcab@gmail.com (A.F.-C.); robustiano.pego.reigosa@sergas.es (R.P.-R.); 3 Centro de Investigación Biomédica en Red en Enfermedades Neurodegenerativas (CIBERNED), Instituto de Salud Carlos III, 28029 Madrid, Spain

**Keywords:** Alzheimer’s disease, autotaxin, lipid signaling, lysophosphatidic acid, neuroinflammation, tau pathology

## Abstract

Alzheimer’s disease (AD) is a progressive neurodegenerative disorder characterized by the accumulation of amyloid-β plaques, tau hyperphosphorylation, and chronic neuroinflammation. Emerging evidence suggests a crucial role of lipid signaling pathways in AD pathogenesis, particularly those mediated by autotaxin (ATX) and lysophosphatidic acid (LPA). ATX, an enzyme responsible for LPA production, has been implicated in neuroinflammatory processes, blood–brain barrier dysfunction, and neuronal degeneration. LPA signaling, through its interaction with specific G-protein-coupled receptors, influences neuroinflammation, synaptic plasticity, and tau pathology, all of which contribute to AD progression. This review synthesizes recent findings on the ATX/LPA axis in AD, exploring its potential as a biomarker and therapeutic target. Understanding the mechanistic links between ATX, LPA, and AD pathology may open new avenues for disease-modifying strategies.

## 1. Introduction

Alzheimer’s disease (AD) is the leading neurodegenerative cause of dementia in developed countries [1]. In the United States, it is estimated that 6.2 million individuals aged 65 and older are living with AD, a number that could reach 13.8 million by 2060 [1]. AD ranks as the sixth-leading cause of death in the US [1]. Clinical manifestations of AD encompass cognitive impairment, psychiatric symptoms, behavioral changes, sleep disturbances, movement disorders, and seizures [2]. It invariably leads to functional impairment, as no treatment has been proven to cure the disease [2].

The major neuropathological features of AD include extracellular deposits of β-amyloid (Aβ), which form amyloid plaques and intracellular accumulation of hyperphosphorylated tau protein in neurofibrillary tangles (NFT) [3]. Additionally, AD has been associated with vascular pathology [4], neuroinflammation [5], oxidative stress [6], and alterations in lipidic metabolism [7,8].

Lipid metabolism has been extensively studied and established as a significant risk factor for the development of AD [9,10,11]. The primary genetic risk factor for developing late-onset AD is the presence of the e4 allele of the apolipoprotein E gene (APOE4). Among the various isoforms of APOE, APOE3 does not appear to influence AD, APOE2 (the least common) may have a protective effect, while APOE4 increases the risk of developing the disease [12,13,14].

The brain is composed of lipids in approximately 60% to 70% by dry weight, which are mainly structural components. However, many of them have cellular signaling properties. In this regard, lysophospholipids are metabolic intermediates derived from membrane phospholipids via hydrolysis. Interestingly, intracellular lysophospholipids are intermediate precursors for the biosynthesis of other lipids, while extracellular lysophospholipids act as signaling molecules. In recent years, lysophosphatidic acid (LPA), one of the most important lysophospholipids, has been studied as a key modulator in several diseases [15].

LPA is a bioactive lipid that acts as a potent extracellular signaling molecule with numerous systemic functions [16], including its involvement in central nervous system (CNS) development. The implication of LPA in brain development is well known [15,17]. Therefore, dysregulation of LPA has been suggested to be involved in several neurological disorders [16,18]. Autotaxin (ATX) is an extracellular enzyme responsible for converting lysophospholipids into LPA, maintaining physiological concentrations of the latter, thus guaranteeing its function as a signaling molecule [18]. In this regard, in recent years the involvement of the ATX/LPA axis has been observed in different neurological diseases, such as AD [19], Parkinson’s disease [20], major depressive disorder [21], neuropathic pain [22], migraine [23], and loss of blood–brain barrier (BBB) integrity [24].

This review explores the role of ATX and LPA signaling in the pathogenesis of AD, highlighting their involvement in neuroinflammation, tau phosphorylation, and β-amyloid metabolism.

## 2. Autotaxin and Lysophosphatidic Acid

ATX, also known as ectonucleotide pyrophosphatase/phosphodiesterase (ENPP2, encoded by the homonym gene), is a secreted lysophospholipase D (lysoPLD) that belongs to the ENPP family [25]. ATX is widely expressed, with the highest mRNA levels detected in the brain, spinal cord, ovaries, lungs, intestines, and kidneys [24].

ATX is synthesized as a preproenzyme requiring post-transcriptional modifications to be active [25]. The ENPP2 gene undergoes alternative splicing and produces four known isoforms (α, β, γ, and δ). ATX β is the canonical, predominant, and most studied form. Interestingly, ATX γ is a “brain-specific” isoform. However, despite the differences in structure, it is currently unclear whether the isoforms are associated with distinct physiopathological conditions or not [25].

LPA (1- or 2-acyl-sn-glycerol 3-phosphate/radyl-glycerol-phosphate), consists of a glycerol backbone, a phosphate group at the sn-3 position, and a hydroxyl group added to a fatty acid chain in sn-1 or sn-2 positions. Different variants are formed depending on the acyl chain length, with 16:0-LPA, 18:2-LPA, and 18:1-LPA being the most abundant in humans. In this regard, the 18:1-LPA form is commonly used in research [16].

ATX primarily produces LPA from extracellular lysophosphatidylcholine (LPC), although it can also degrade lysophosphatidylethanolamine and lysophosphatidylserine with less affinity [26,27]. It is well established that ATX is the main source of synthesized LPA [28]. Due to rapid clearance from circulation through liver degradation [29], the main biological effects of the ATX/LPA axis result from LPA signaling. Extracellular LPA can be found in various biological fluids, such as plasma, cerebrospinal fluid (CSF), saliva, tears, and aqueous humor [26,30]. The highest serum levels of LPA are produced by platelets during clotting [31], when concentrations of 10–15 μM in serum can be reached [30]. In this context, concentrations of less than 1 μM in plasma are considered “normal”; and even lower levels in cerebrospinal fluid (CSF) [30].

LPA has been shown to play a role in numerous physiological functions, including blood pressure regulation, cell proliferation and survival, calcium mobility, and angiogenesis, among others [32]. It has also been implicated in pathological processes, such as different types of cancer [33,34,35,36,37,38,39,40,41], rheumatologic diseases [42,43], pulmonary fibrosis [44,45], psychiatric disorders, and neurological disorders [23,46,47,48,49].

In addition to extracellular production, there is also intracellular production of LPA through pathways including monoacylglycerol kinase (MAGK), PA-PLA1 or PA-PLA2, glycerophosphate acyltransferase (GPAT) synthesis, and the oxidative modification of low-density lipoprotein (LDL) [16]. Intracellular LPA functions as a substrate for glycerolipid synthesis and is unlikely to serve as an extracellular mediator [18].

## 3. LPA Signaling in the Central Nervous System

LPA acts through six specific G protein-coupled receptors (GPCRs) to maintain homeostasis in relevant processes such as cell proliferation, migration, or cytoskeletal reorganization [16,30,50,51]. These six identified receptors (LPA1 to LPA6) [52] differ in their G-protein coupling, downstream signaling pathways, tissue distribution, and physiological functions (Table 1). They are also encoded by different genes (*LPAR1–6)* [53].

The first three LPA receptors (LPA1–3) belong to the endothelial differentiation gene (Edg) subfamily and have been studied in more detail, whereas the last three (LPA4–6) have been more recently discovered, and belong to the phylogenetically distant non-Edg subfamily of receptors [54]. All these receptors are 7-transmembrane GPCRs and exhibit high affinity for LPA. Consequently, these GPCRs are coupled with one or several heterotrimeric G-proteins from the four existing classes (Gα12/13, Gαq/11, Gαi/o, and Gαs), which can initiate a wide range of downstream pathways (Table 1) [52].

**Figure 1:**
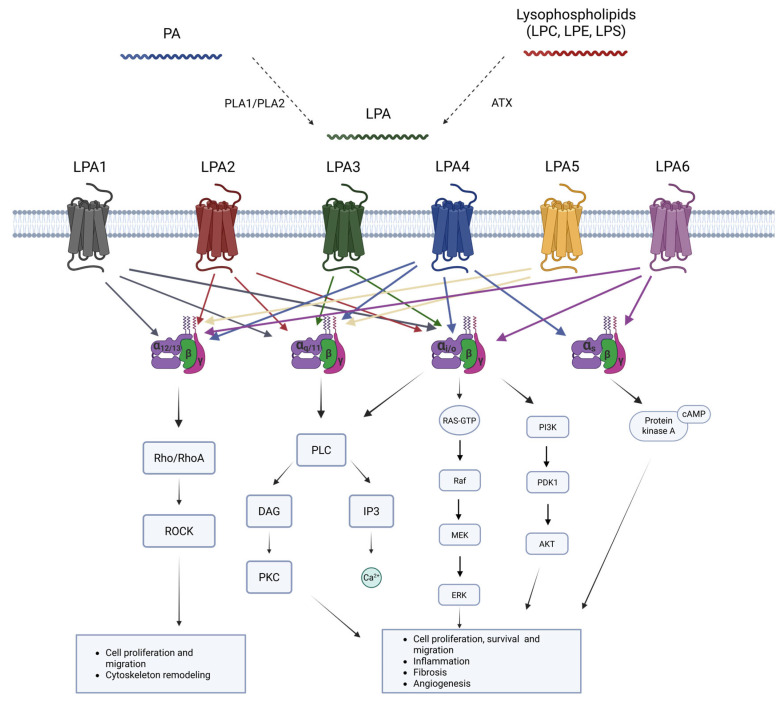
Summary of LPA synthesis routes and signaling pathways through its GPCR receptors, inducing several cellular responses. Abbreviations: ATX—Autotaxin; cAMP—Cyclic adenosine monophosphate; DAG—Diacylglycerol; GPCRs—G-coupled protein receptors; IP3—inositol trisphosphate; LPA—Lysophosphatidic acid; LPC—Lysophosphatidylcholine; LPE—Lysophosphatidylethanolamine; LPS—Lysophosphatidylserine; MAPK—Mitogen-activated kinase; MEK—Mitogen-activated protein kinase kinase; PA—phosphatidic acid; PDK1—phosphoinositide-dependent kinase; PI3K—phosphatidylinositol 3-kinase; PLC—Phospholipase C; PKC—Protein kinase C; RhoA—Ras homolog family member A; ROCK—Rho-associated kinase.

As mentioned, LPA receptors are widely distributed throughout several systems in the organism, accounting for their equally diverse biological functions. Among these systems, the CNS stands out for the greater significance of LPA signaling [55,56]. LPA, acting through its receptors LPA1–6, has a main role in the early stages of mouse brain development and neurogenesis [57,58,59]. Moreover, LPA and its analogs have been observed to promote neurotrophic effects and neurite outgrowth in cultured cells [60]. Furthermore, LPA has been suggested to play a role in synaptic plasticity and modulation, and it could also be involved in the development of neuropathic pain [61]. LPA receptors are also found in CNS glial cells, where they play a role in mediating the development of oligodendrocytes, activating microglia and potentially inducing neuronal differentiation [17,32,51,55,62]. Furthermore, these receptors may have a regulatory function in activating microglia-mediated neuroinflammation through mitogen-activated protein kinase (MAPK) and AKT activation [63]. A summary of LPA metabolism and its receptors can be found in Figure 1.

In addition to its physiological functions, LPA signaling is involved in the pathogenesis of diseases, including neurological diseases (such as AD, Parkinson’s disease, and Huntington’s disease) [64,65], heart and vascular conditions (for instance, heart failure) [66,67], respiratory pathologies (i.e., pulmonary fibrosis or pulmonary hypertension [68]), gastrointestinal afflictions [69], and different types of cancer [70]. Under pathological conditions, LPA signaling can undergo significant alterations, thus contributing to disease progression [30]. Different LPA receptors mediate pathogenesis-related processes in the CNS in several ways, as summarized in Table 2. Therefore, studying them as potential biomarkers or therapeutic targets proves to be a promising strategy in the search for new drugs or diagnostic tests.

### 3.1. LPA1

LPA1, being the first receptor to be discovered [100,101], is also the most extensively studied. As with LPA2 and LPA3, it belongs to the family of the endothelial differentiation gene (Edg) receptors. Initially named Vzg-1, this receptor was isolated from the ventricular zone of a neuroblast cell line, where it is highly expressed [100]. LPA1 is the most prevalent receptor in human and mouse brain tissue, and it can be coupled with three of the Gα proteins, Gαi/o, Gαq/11, and Gα12/13, thereby initiating downstream signaling cascades through phospholipase C, MAPK, protein kinase B (PKB, also known as Akt), and Rho [30]. Recently, the LPA1 structure was elucidated, providing new insights into its activation mechanisms [102]. Its *K_d_* values vary, depending on the LPA species, between 0.87 and 69 nM [103,104,105]. Beyond the CNS, LPA1 presents a broad distribution, promoting a diverse range of cellular responses, including cell proliferation and survival, and Ca^2+^ mobilization, among others [32,106].

Extensive research has been conducted concerning the role of LPA1 in neurodevelopment, as it participates in a wide range of processes. Some notable examples include its involvement in regulating myelination during the postnatal period, as well as in developing white matter tracks [107,108], in cortical development [109,110,111], and in cell migration [112]. Additionally, LPA1 plays an essential role in the development of the peripheral nervous system by controlling the function and growth of Schwann cells [113]. In this regard, mutant mouse models lacking this specific receptor show a significant impairment in hippocampal neurogenesis [114]. Moreover, descendants of the *Lpar1−/−* line have only a 50% survival rate due to impaired suckling behavior, and the surviving half display anomalies such as reduced body size, cranial dysmorphism, and increased apoptosis in the Schwann cell population of the sciatic nerve [115,116]. In recent years, new knock-in mouse models have been developed, replacing one of the wild-type alleles of *LPA1* with a new mutant (*Lpar1-EGFP* and LPA1-LacZ). This has enabled new in situ studies of this receptor’s functions, showing that LPA1 is highly expressed in oligodendrocytes [117,118]. Overall, the evidence gathered strongly suggests that LPA1 is a crucial modulator of neurogenesis.

LPA1 has also been found to play a role in neuropathic pain [22]. LPA1 and LPA3 receptor-deficient mice showed absent allodynia in paclitaxel-induced neuropathy [119], neuropathic pain due to diabetic neuropathy [120], and central post-stroke pain [121]. Accordingly, mice treated with LPA1 and LPA3 antagonists showed reversed abnormal pain behaviors [119,120,121].

### 3.2. LPA2

LPA2 is another well-characterized receptor [122] that promotes neurogenesis by inducing neurite formation, like LPA1 [123], with some overlapping functions. Its *K_d_* is approximately 64 nM [105]. LPA2 exhibits a more diffuse expression pattern in the developing brain [30,124]. However, LPA2 null mice do not exhibit any obvious differences in phenotype [125], in contrast to those lacking LPA1, which might suggest a redundant effect of LPA2 in the developing mouse brain. The lack of both receptors in double-knockout mice showed reduced LPA-induced responses in embryonic fibroblasts [125].

Interestingly, it has been reported that an upregulation of LPA2 exists in the adult mouse brain after traumatic brain injury (TBI) in the astrocytes and neurons [126,127]. This upregulation has also been observed in astrocytes after spinal cord injury [127], as well as after ischemia in the inner layers of rat retina, suggesting compensatory mechanisms to promote cell survival [128].

### 3.3. LPA3

LPA3 was initially studied for its role in embryo implantation [129]. In recent years, it has also been studied as a potential biomarker and therapeutic target in ovarian cancer [130].

LPA3 plays an essential role in the formation of neuronal networks [131], as it can mediate axonal branching (in contrast to other LPA receptors), and it also plays a critical role regulating the expression of the antioxidant enzymes that eliminate reactive oxygen species (ROS) [132]. Likewise, it has been found to increase its expression following brain injury [127]. More recently, a highly specific LPA3 agonist, ADS024-IPA, has been shown to improve outcomes when given orally to different animal models of neuroinflammatory disease, suggesting a potential role as a prophylaxis or treatment for these pathologies [133]. LPA is a poor ligand for LPA3 [134].

As previously mentioned, LPA3 may also participate in the regulation of neuropathic pain, like LPA1, and treatment with LPA1 and LPA3 antagonists was useful in mice as a potential chronic pain treatment [22,119,120,121].

### 3.4. LPA4

LPA4 was the first receptor to be characterized with a structural difference from the previously discovered LPA receptors, as it is a non-Edg receptor [54]. LPA4 has a specific binding affinity to 18:1-LPA with a *K_d_* value of 44.8 nM to 100 nM, but not to other lysophospholipids and related lipids, such as S1P and SPC [104,105,135].

This receptor can induce neurite retraction and stress fiber formation through the Rho/ROCK pathways [30,136,137]. Intracellularly, LPA4 increases concentrations of Ca^2+^ and cAMP, the latter serving as a complementary effect against the cAMP attenuating activities of LPA1–3 [137]. LPA4 also mediates ROCK-dependent cell aggregation and N-cadherin-dependent cell adhesion [136]. It is noteworthy that LPA4 negatively regulates cell motility and inhibits the effects of LPA1 on cell migration [138]. The LPA4 axis is required for bipolar morphogenesis and radial migration, modifying the actin cytoskeleton of newborn cortical neurons [139]. Interestingly, LPA4 null mouse lines appear mostly normal [138], like LPA2 null lines.

### 3.5. LPA5

Discovered in 2006, LPA5 is another non-Edg LPA receptor, which binds to Gαq and Gα12/13, Gαi, and Gαs [16,30,140]. Its *K_d_* ranges from 6 to 89 nM [104,105,140]. It is involved in neurite retraction mediated by Gα12/13 and Rho [137].

Additionally, LPA5 is speculated to be involved in nociception and pain hypersensitivity mechanisms, as well as in anxiety-related and motivation-driven behaviors [141]. Moreover, it is highly expressed in the dorsal root ganglia [140], thus suggesting its possible involvement in neuropathic pain. This was confirmed in *Lpar5* null mice, which had decreased pain and a lower level of phosphorylated cyclic AMP response element binding protein (pCREB) expression [95]. The use of the LPA5 antagonist AS2717637 in rodents has also shown analgesic effects [142]. The use of another LPA5 antagonist, TCLPA5, has been shown to disrupt the pro-inflammatory effects of this receptor, interfering with microglia polarization [143].

### 3.6. LPA6

LPA6, the most recently discovered receptor, is predominantly expressed in brain capillary endothelial cells, where the LPA–LPA6–G12/13–Rho pathway plays a part in BBB permeability [98]. Nevertheless, much remains unknown about the role of this receptor in the CNS. Its *K_d_* has not been accurately determined yet, probably due to low affinity or the rapid off rates of LPA [105]. In addition, a recent study demonstrated that LPA6 expression in oligodendrocytes could be considered a negative regulator of myelination during CNS development [99].

## 4. ATX/LPA Axis and Alzheimer’s Disease

It is well known that the accumulation of Aβ is the hallmark of AD [144]. The increasing deposits of Aβ produce neurotoxicity in the CNS [145], contributing to tau hyperphosphorylation [146], and resulting in neuronal cell death. Recently, a clear correlation between dysfunctional LPA signaling and the pathogenesis of AD has been established [19], along with alterations in other bioactive lipids, such as S1P [8,62].

Furthermore, substantial evidence supports a significant degree of oxidative damage to lipids and proteins throughout the progression of AD [147]. CSF lipoproteins are particularly sensitive to oxidation, becoming neurotoxic in their oxidized state [148]. Interestingly, LPA is the main bioactive component of oxidized low-density lipoprotein (oxLDL), a high-cardiovascular risk molecule [149]. Regarding AD, oxLDL-LPA induces an elevation in Aβ production by activating the LPA/BACE1/APP axis [150].

LPA can induce the activation of delta protein kinase C (PKCδ) [151], triggering a downstream signaling cascade through consecutive phosphorylation of mitogen-activated protein kinase kinase (MEK), MAPK, p90RSK, and finally cyclic AMP response element binding protein (CREB) [150]—a transcription factor known to upregulate BACE1 expression by binding to its gene promoter region when active [152]. BACE1 [153], also known as beta-site amyloid precursor protein cleaving enzyme 1 or simply beta-secretase, together with gamma-secretase [154], are the secretases directly responsible for producing Aβ from amyloid precursor protein (APP) [155].

Cholesterol is another lipidic molecule that has been linked to the pathogenesis of AD, also through its contribution to the accumulation of Aβ [156]. Lipid rafts, domains in the plasma membrane [157] enriched in cholesterol and sphingolipids, play a role in promoting the interaction between APP and BACE1 [158]. OxLDL is one of the major cholesterol carriers and contributes to the increase in lipid raft formation along the plasma membrane in AD through a redox-imbalance process [159]. The oxidative stress caused by oxLDL triggers glutathione (GSH) peroxidase activity, leading to the depletion of GSH [160]. In the absence of GSH, the enzyme sphingomyelinase increases its activity, likewise contributing to lipid raft formation, and consequently increasing Aβ formation [159].

**Figure 2:**
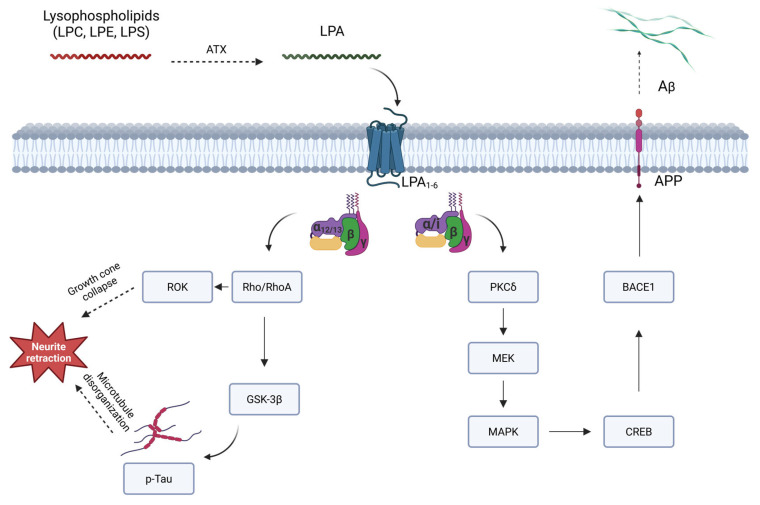
Schematic representation of the role of LPA in AD pathogenesis. LPC is transformed by ATX into LPA, which interacts with GPCRs in the cell membrane. Then, the Rho/RhoA pathway leads to hyperphosphorylation of tau, microtubule disorganization, and actomyosin contraction, ultimately producing neurite retraction. Through the MAPK pathway, BACE1 is overexpressed, cleaving APP and producing Aβ monomers. Abbreviations: AD—Alzheimer’s disease; APP—Amyloid precursor protein; ATX—Autotaxin; BACE1—β-site APP-cleaving enzyme 1 (β-secretase); CREB—Cyclic AMP response element binding protein; GPCRs—G-coupled protein receptors; GSK-3β—Glycogen synthase kinase-3 β; LPA—Lysophosphatidic acid; LPC—Lysophosphatidylcholine; LPE—Lysophosphatidylethanolamine; LPS—Lysophosphatidylserine; MAPK—Mitogen-activated protein kinase; PKC—Protein kinase C.

As mentioned, post-translational modifications of tau are also key mediators in the pathogenesis of AD [146]. Tau is an essential component for the proper functioning of microtubules [161]. Hyperphosphorylated tau forms aggregates of paired helical filaments, and the extreme aggregation of these filaments results in the neurofibrillary tangles responsible for the pathogenesis of AD [162]. In this sense, LPA can contribute to tau phosphorylation through the action of glycogen synthase kinase-3β (GSK-3β) [163,164]. This kinase is highly expressed in the brain and, under normal conditions, plays a role in axonal growth during neurodevelopment [165]. However, dysregulations of this enzyme are associated with the vast majority of pathological processes in AD [166]. The most probable mechanism involves the LPA/RhoA/ROCK pathway, as observed in other related cases [167]. In fact, it has already been documented that Rho kinases are involved in tau hyperphosphorylation [168]. Furthermore, GSK-3β inhibitors have been shown to block tau hyperphosphorylation and to improve tauopathies [169], confirming the involvement of this enzyme in the process. These mechanisms are represented in Figure 2.

### 4.1. ATX and LPA as Potential Biomarkers in Alzheimer’s Disease

As mentioned earlier, LPA mediates neurite retraction during neuronal development. However, anomalies can result in an excessive amount of neurite retraction, leading to diseases in adult individuals [164]. LPA-induced tau hyperphosphorylation by GSK-3β is a potential cause of pathological neurite retraction and is linked with AD [170]. While inhibitors of GSK-3β improve neurite retraction, they do not block it completely [163], indicating the involvement of other important factors. For example, kinases, such as p38 MAPK [171] and CDK5 [172], are also known to be linked to this process.

These interactions are further supported by the research on LPA levels in CSF. Various isoforms of this lysophospholipid were analyzed in CSF, demonstrating a significant association between their levels and concentrations of classical AD biomarkers, such as Aβ-42, p-tau, and total tau [173].

LPA1 is also highly relevant in the context of AD. As previously mentioned, various models of LPA1-null mice have provided insights into the functions of this receptor. Behavioral studies have indicated that, in the absence of LPA1, mice exhibit anxiety, memory impairment, and motor abnormalities [174,175], highlighting the role of this receptor in the correct functioning of these processes. An expression profiling of circular RNAs in CSF from AD patients revealed an upregulation of *Circ-LPAR1* expression, suggesting its potential as a biomarker for AD [176]. Moreover, *Circ-LPAR1* has been suggested to be involved in inducing neuronal apoptosis, inflammation, and oxidative stress [177].

In a recent study [178] carried out on a triple transgenic mouse model of AD, LPA1 activity was found to be increased in the corpus callosum, motor cortex, hippocampal CA1 area, and striatum. The authors postulated that this increase could be related to adaptations during the development of the 3xTg-AD mice, increasing the demand for the LPA endogenous neurotransmitter and elevating the levels of lipid precursors.

Moreover, ATX is also associated with AD. Studies have observed significantly higher expression of ATX in the frontal cortex of patients suffering from AD [179]. ATX concentrations in CSF can serve as a biomarker of brain metabolic dysfunction, as they have been found to be increased in patients with AD and mild cognitive impairment (MCI). This elevation correlates with hypometabolism in the prefrontal cortex and mesial temporal lobes in positron emission tomography (PET) scans, as well as with concentrations of classical AD biomarkers [180]. However, further investigation into the underlying mechanisms is needed.

Another known risk factor for AD is TBI [181]. TBI causes BBB dysfunction [182], leading to an increased presence of LPA in the CNS. Therefore, there is an amplification of all the downstream consequences mentioned above, creating a positive feedback loop, as high concentrations of LPA also contribute to the permeabilization of the BBB through the Rho/ROCK/MMP-9 or uPA pathways [183].

### 4.2. Advances and Challenges in Therapy Targeting the ATX/LPA Axis

In addition to its roles earlier discussed, LPA1 serves as an important mediator in adult hippocampal neurogenesis [184], the hippocampus being a key region for memory, and therefore highly relevant in the context of AD [185]. Studies have shown that LPA administration can decrease cocaine-contextual memory in models of cocaine addiction by stimulating hippocampal neurogenesis [184]. Further research is needed on the topic of LPA-induced adult hippocampal neurogenesis to explore potential applications for the development of new strategies against AD. Indeed, the creation of new therapies involving LPA1 and other receptors may not only serve as valuable tools for functional studies but may open avenues for the design of new treatments [186,187,188,189].

As mentioned earlier, the hippocampus is a key region for memory. Pathological concentrations of LPA can induce apoptosis/necrosis of the hippocampal neurons [190]. However, LPA is also necessary for the proper functioning of the hippocampus. Inhibitors of LPA receptors have shown that LPA regulates cognition and emotion [191]. Also, the administration of external LPA [191] or antidepressants that behave as agonists of LPA receptors [192] can act as potential treatments favoring the neuroprotection of the hippocampus.

Neuroinflammation has neurotoxic effects on the CNS and is closely related to the pathogenesis of AD [5]. Markers associated with the immune system have been found in the brain and biological fluids of patients with this disorder [193,194]. Microglial activation correlates with NFT and amyloid plaque load in AD and MCI [195]. More recently, the inflammasome has also been suggested to play a role in the disease, perpetuating chronic microglial-induced inflammation of the CNS and exacerbating tau pathology [196]. Aβ and NFT can lead to activation of Toll-like receptors and the NRLP3 inflammasome, inducing microglia to generate pro-inflammatory mediators [196]. Systemic inflammation can induce aberrant LPA signaling, which is known to cause microglia polarization and, consequently, neuroinflammation through MAPK-dependent pathways [197]. In addition, LPA can promote BV-2 and primary murine microglia to convert into a pro-inflammatory M1-like phenotype [143]. In mice with experimental autoimmune encephalomyelitis, increased ATX and LPA levels were found in plasma and spinal cord. Furthermore, ATX was found to be expressed in CD11b+ cells (microglia and macrophages), and genetic deletion of ATX from these cells improved the progression of the disease [198].

## 5. Future Directions: ATX/LPA Axis as a Potential Therapeutic Target

ATX inhibition has enormous potential, including possible applications in pathologies like brain cancer, pruritus, pain-related dysfunctions, or neurodegenerative diseases [199], but we are still beginning to explore this field and further research is needed. Two oral ATX inhibitors, GLPG1690 and FTP-180, are currently on trial for idiopathic pulmonary fibrosis [200,201] and ziritaxestat, another orally administered ATX inhibitor, for systemic sclerosis [202]. On the other hand, a phase three clinical trial for ziritaxestat has failed to improve outcomes in patients with idiopathic pulmonary fibrosis [203].

New ATX inhibitors have been designed recently, of which the non-zinc-binding ATX inhibitor BIO-32546 is selective, orally bioavailable, and capable of penetrating into the brain, and has demonstrated in vivo efficacy for acute pain [204]. More recently, cannabinoids have been used as ATX inhibitors, the most potent of them being MEY-003, which has been found to inhibit in vitro (but it has potential to be orally bioavailable) both ATX β and ATX γ, making it a possible therapeutic agent to treat neurological disorders [205]. Another ATX inhibitor derived from ursodeoxycholic acid has also been recently developed, which targets the hydrophobic tunnel and active site of the phosphodiesterase domain [206]. Fingolimod, a well-known oral drug used to treat multiple sclerosis, whose main mechanism of action is the modulation of the S1P receptors, has been found to inhibit ATX [207,208]. In mice, this has resulted in peripheral nerve regeneration after sciatic nerve crush, particularly in wildtype C57BL/6 and Foxn1 (-/-), but not in Rag1 (-/-) [209]. Furthermore, the ATX inhibitor PF-8380, with potential oral bioavailability, improved neuroinflammation in mouse models of hepatic encephalopathy, reducing levels of LPA 18:0 in the cerebral cortex, hippocampus, brain edema, and plasma, consequently improving motor and cognitive functions [210]. Currently, no ATX inhibitor has been tested specifically for AD.

The actions of LPA1 have also been targeted for therapeutic strategies. The effects of gintonin, a glycolipoprotein fraction of ginseng that is enriched in LPA [211], are also mediated by LPA1. Gintonin has been the focus of recent research because it is a natural, easy-to-obtain product with several potential therapeutic applications, there being strong evidence of its neuroprotective character [212]. Its administration seems safe and might improve the condition of cognitively impaired elderly people [213]. Gintonin could allegedly promote non-amyloidogenic processing to restore brain function when administered orally in mice with AD (AβPPswe/PSEN-1 double Tg mice) [214]. Gintonin-mediated LPA can also activate the LPA1/BDNF/TrkB/Akt pathway to attenuate oxidative damage caused by iodoacetic acid [215], a possible risk factor of AD. Its therapeutic action could also be explained through its possible role in neuronal morphological changes and migration by activating the LPA1/3 receptors [216]. Moreover, it can increase BBB permeability, improving the delivery of drugs into the CNS [217], one of the largest obstacles for the treatment of neurological diseases. Specifically, the intravenous coadministration of gintonin with donepezil, a cognition-improving drug used in AD [218], enhances donepezil brain delivery through gintonin action over the LPA1/3 and VEGF receptors [219].

There are also monoclonal antibodies against LPA in the early phases of preclinical studies, such as Lpathomab and its derivative, called 504B3, which shows potential for improving patient outcomes following TBI when given intravenously in C57BL/6 J mice [220,221]. B3 was also tested both in vitro and in vivo, by subcutaneous administration in zebrafish, to analyze its capacity to improve spinal cord injury outcomes. It has been shown to effectively block LPA1–3 receptors, to decrease inflammation in scar tissue, and to promote neuronal cell survival and synaptic density [222].

As of today, no clinical trials have been initiated using ATX/LPA axis inhibitors for the treatment of AD.

## 6. Final Remarks

This review highlights the pivotal role of the ATX/LPA axis signaling pathway in the pathophysiology of AD. This signaling cascade, through both direct and indirect mechanisms, emerges as a crucial player in several pathological processes, particularly contributing to neurodegeneration.

The components comprising the ATX/LPA axis exhibit promising potential not only as contributors to our understanding of AD but as essential elements for potential diagnostic biomarkers and therapeutic targets. Early diagnosis is crucial for implementing effective treatment strategies and potentially slowing down the degenerative processes in the central nervous system.

Further research focused on the roles of ATX and LPA in neurological diseases, particularly AD, is imperative. Deeper insights into the intricate interactions within the central nervous system will not only expand our understanding of the pathophysiology of AD but open avenues for the development of targeted therapeutic approaches.

In conclusion, the ATX/LPA axis emerges as a key signaling cascade in the complex landscape of AD. Its multifaceted involvement in pathological processes positions it as a promising area for future research, offering potential breakthroughs in both diagnostics and therapeutics, ultimately aiming to enhance our ability to tackle this challenging neurodegenerative disorder.

## Figures and Tables

**Table 1:** LPA receptors signaling pathways, functions, and tissue distribution. Abbreviations: cAMP—Cyclic adenosine monophosphate; FAK—focal adhesion kinase; LPA—Lysophosphatidic acid; MAPK—Mitogen-activated protein kinase; PI3K—phosphatidylinositol 3-kinase; PKA—Protein kinase A; PKC—Protein kinase C; PLC—Phospholipase C; RhoA—Ras homolog family member A; ROCK—Rho-associated kinase.

Receptor	G Proteins	Main Signaling Pathways	Functions	Tissue Distribution
LPA_1_	Gi, Gq, G12/13	PI3K/Akt, RhoA/ROCK, PLC/Ca^2+^	Cell migration, proliferation, fibrosis, nervous system development.	Brain, heart, lung, skin, adipose tissue.
LPA_2_	Gi, Gq, G12/13	PI3K/Akt, MAPK, RhoA, FAK	Cell survival, immune modulation, tissue repair.	Immune cells, lungs, intestines.
LPA_3_	Gi, Gq,	MAPK, PKC/Ca^2+^, RhoA/ROCK	Angiogenesis, metastasis, reproductive functions.	Ovaries, uterus, brain, prostate.
LPA_4_	Gs, Gq, Gi, G12/13	cAMP/PKA, PLC/Ca^2+^, RhoA	Cell shape regulation, vascular development.	Embryonic tissues, lung, heart.
LPA_5_	Gq, G12/13	cAMP/PKA, RhoA/ROCK	Pain modulation, platelet activation, immune response.	Brain, spleen, immune cells.
LPA_6_	Gs, Gi, G12/13	RhoA/ROCK, Cytoskeletal remodeling	Cell contraction, vascular permeability, inflammation.	Skin, lung, heart.

**Table 2:** LPA receptors and their role in pathological processes. Abbreviations: LPA—Lysophosphatidic acid; NLRP3—NLR family pyrin domain containing 3; NSCs—neural stem cells; pCREB—phosphorylated cyclic AMP response element binding protein; Rho: Ras homolog family member; ROCK—Rho-associated kinase; TNF—tumor necrosis factor.

Receptor	Pathological Process	Functions	References
LPA1	Neurodegeneration	Synaptic dysregulation, apoptotic cell death in null mutants.	[71]
	Ischemic stroke	Apoptosis of PC12 cells through mitochondrial dysfunction.	[72]
	Focal cerebral ischemia	Activation of the NLRP3 inflammasome and neuroinflammation.	[73,74]
	Neuropathic pain	Expression of pain-related genes/proteins and demyelination.	[75,76]
		Modulation of synaptic excitatory transmission.	[61]
	Intracerebral hemorrhage	Neuroinflammation.	[77]
	Posthemorrhagic hydrocephalus	Ependymal cell ciliary dysfunction, reduced motility, damage, and death.	[78]
	Glioblastoma	Tumor survival, growth, and migration.	[79,80]
	Multiple sclerosis	Macrophage-mediated neuroinflammation.	[81]
	Amyotrophic lateral sclerosis	Motor neuron dysregulation of intrinsic membrane excitability and degeneration.	[82,83]
	Epileptic seizures	Regulates the induction and division of reactive NSCs in the dentate gyrus.	[84]
	Spinal cord injury	Secondary damage by demyelination and functional deficits.	[85]
	Neuropsychiatric disorders		
	Affective disorders	Thrombospondin-1 production in astrocytes.	[86]
		Inhibition of the TNFα-induced apoptosis of HT22 hippocampal cells improves depression disorders.	[87]
	Anxiety-like disorders	Absence of the receptor equals GABAergic hippocampal interneuron deficit.	[88]
		Exacerbates stress behavior under chronic stress conditions.	[89,90]
LPA2	Ischemic stroke	Same mechanism as LPA1.	[72]
	Amyotrophic lateral sclerosis	Accelerates disease onset and neurological deficit in the early stages; however, extends lifespan on the long term.	[91]
	Spinal cord injury	Demyelination and microglia-induced cytotoxicity.	[92]
LPA3	Neuropathic pain	Pain memory and related neural plasticity.	[76]
	Posthemorrhagic hydrocephalus	Same mechanism as LPA1.	[78]
	Affective disorders	Thrombospondin-1 production in astrocytes.	[86]
LPA4	Lumbar spinal stenosis	Gα12/13–Rho–ROCK2-induced apoptosis, DNA damage, and oxidative stress in spinal cord neurons.	[93]
LPA5	Focal cerebral ischemia	Microglial activation and pro-inflammatory responses.	[94]
	Neuropathic pain	Contributes through central pCREB activation, with different mechanisms from LPA1.	[95]
		Is involved in hyperalgesia through Aδ-fibers, but not in demyelination.	[96]
	Systemic inflammation diseases	Polarizes microglia toward a pro-inflammatory phenotype.	[97]
LPA6	Cerebral edema	LPA6–G12/13–Rho pathway may lead to enhanced permeability of the BBB.	[98]
	Multiple sclerosis	Persisting expression in oligodendrocytes inhibits myelin repair.	[99]
	Lumbar spinal stenosis	Same mechanism as LPA4.	[93]

## Abbreviations

The following abbreviations are used in this manuscript:

Aβ	β-amyloid
AD	Alzheimer’s disease
APOE	Apolipoprotein E
APP	Amyloid precursor protein
ATX	Autotaxin
BACE1	Beta-site amyloid precursor protein cleaving enzyme 1
BBB	Blood–brain barrier
cAMP	Cyclic adenosine monophosphate
CDK5	Cyclin-dependent kinase 5
CNS	Central nervous system
CREB	cAMP response element binding protein
CSF	Cerebrospinal fluid
Edg	Endothelial differentiation gene
ENPP2	Ectonucleotide pyrophosphatase/phosphodiesterase
GPCRs	G protein-coupled receptors
GSH	Glutathione
GSK-3β	Glycogen synthase kinase-3β
LPA	Lysophosphatidic acid
LPC	Lysophosphatidylcholine
lysoPLD	Lysophospholipase D
MAPK	Mitogen-activated protein kinase
MCI	Mild cognitive impairment
MEK	Mitogen-activated protein kinase kinase
MMP-9	Matrix metallopeptidase 9
NFT	Neurofibrillary tangles
NRLP3	NLR family pyrin domain containing 3
oxLDL	Oxidized low-density lipoprotein
pCREB	Phosphorylated cyclic AMP response element binding protein
PET	Positron emission tomography
PKB	Protein kinase B
PKCδ	Protein kinase C delta
RhoA	Ras homolog family member A
ROCK	Rho-associated kinase
ROS	Reactive oxygen species
S1P	Sphingosine-1-phosphate
SPC	Sphingosylphosphorylcholine
TBI	Traumatic brain injury
uPA	Urokinase plasminogen activator
VEGF	Vascular endothelial growth factor

## References

[1] Alzheimer’s Association (2021). 2021 Alzheimer’s Disease Facts and Figures. Alzheimer’s Dement..

[2] McDade E.M. (2022). Alzheimer Disease. Contin. Lifelong Learn. Neurol..

[3] Gallardo G., Holtzman D.M. (2019). Amyloid-β and Tau at the Crossroads of Alzheimer’s Disease. Adv. Exp. Med. Biol..

[4] Custodia A., Ouro A., Romaus-Sanjurjo D., Pías-Peleteiro J.M., de Vries H.E., Castillo J., Sobrino T. (2022). Endothelial Progenitor Cells and Vascular Alterations in Alzheimer’s Disease. Front. Aging Neurosci..

[5] Heneka M.T., Carson M.J., Khoury J.E., Landreth G.E., Brosseron F., Feinstein D.L., Jacobs A.H., Wyss-Coray T., Vitorica J., Ransohoff R.M. (2015). Neuroinflammation in Alzheimer’s Disease. Lancet Neurol..

[6] Huang W.J., Zhang X., Chen W.W. (2016). Role of Oxidative Stress in Alzheimer’s Disease (Review). Biomed. Rep..

[7] Crivelli S.M., Giovagnoni C., Visseren L., Scheithauer A.L., de Wit N., den Hoedt S., Losen M., Mulder M.T., Walter J., de Vries H.E. (2020). Sphingolipids in Alzheimer’s Disease, How Can We Target Them?. Adv. Drug Deliv. Rev..

[8] Custodia A., Romaus-Sanjurjo D., Aramburu-Núñez M., Álvarez-Rafael D., Vázquez-Vázquez L., Camino-Castiñeiras J., Leira Y., Pías-Peleteiro J.M., Aldrey J.M., Sobrino T. (2022). Ceramide/Sphingosine 1-Phosphate Axis as a Key Target for Diagnosis and Treatment in Alzheimer’s Disease and Other Neurodegenerative Diseases. Int. J. Mol. Sci..

[9] Ahmed H., Wang Y., Griffiths W.J., Levey A.I., Pikuleva I., Liang S.H., Haider A. (2024). Brain Cholesterol and Alzheimer’s Disease: Challenges and Opportunities in Probe and Drug Development. Brain.

[10] Kunkle B.W., Grenier-Boley B., Sims R., Bis J.C., Damotte V., Naj A.C., Boland A., Vronskaya M., van der Lee S.J., Amlie-Wolf A. (2019). Genetic Meta-Analysis of Diagnosed Alzheimer’s Disease Identifies New Risk Loci and Implicates Aβ, Tau, Immunity and Lipid Processing. Nat. Genet..

[11] Haney M.S., Pálovics R., Munson C.N., Long C., Johansson P.K., Yip O., Dong W., Rawat E., West E., Schlachetzki J.C.M. (2024). APOE4/4 Is Linked to Damaging Lipid Droplets in Alzheimer’s Disease Microglia. Nature.

[12] Huang Y., Mahley R.W. (2014). Apolipoprotein E: Structure and Function in Lipid Metabolism, Neurobiology, and Alzheimer’s Diseases. Neurobiol. Dis..

[13] Yamazaki Y., Zhao N., Caulfield T.R., Liu C.C., Bu G. (2019). Apolipoprotein E and Alzheimer Disease: Pathobiology and Targeting. Nat. Rev. Neurol..

[14] Giri M., Shah A., Upreti B., Rai J.C. (2017). Unraveling the Genes Implicated in Alzheimer’s Disease (Review). Biomed. Rep..

[15] Tan S.T., Ramesh T., Toh X.R., Nguyen L.N. (2020). Emerging Roles of Lysophospholipids in Health and Disease. Prog. Lipid Res..

[16] Geraldo L.H.M., de Sampaio Spohr T.C.L., do Amaral R.F., da Fonseca A.C.C., Garcia C., de Almeida Mendes F., Freitas C., Fabio dosSantos M., Lima F.R.S. (2021). Role of Lysophosphatidic Acid and Its Receptors in Health and Disease: Novel Therapeutic Strategies. Signal Transduct. Target. Ther..

[17] Yung Y.C., Stoddard N.C., Mirendil H., Chun J. (2015). Lysophosphatidic Acid Signaling in the Nervous System. Neuron.

[18] Okudaira S., Yukiura H., Aoki J. (2010). Biological Roles of Lysophosphatidic Acid Signaling through Its Production by Autotaxin. Biochimie.

[19] Ramesh S., Govindarajulu M., Suppiramaniam V., Moore T., Dhanasekaran M. (2018). Autotaxin–Lysophosphatidic Acid Signaling in Alzheimer’s Disease. Int. J. Mol. Sci..

[20] Yang X., Zhao E.Y., Zhuang W., Sun F., Han H., Han H., Lin Z., Pan Z., Qu M., Zeng X. (2015). LPA Signaling Is Required for Dopaminergic Neuron Development and Is Reduced through Low Expression of the LPA1 Receptor in a 6-OHDA Lesion Model of Parkinson’s Disease. Neurol. Sci..

[21] Itagaki K., Takebayashi M., Abe H., Shibasaki C., Kajitani N., Okada-Tsuchioka M., Hattori K., Yoshida S., Kunugi H., Yamawaki S. (2019). Reduced Serum and Cerebrospinal Fluid Levels of Autotaxin in Major Depressive Disorder. Int. J. Neuropsychopharmacol..

[22] Ueda H. (2020). LPA Receptor Signaling as a Therapeutic Target for Radical Treatment of Neuropathic Pain and Fibromyalgia. Pain Manag..

[23] Ouro A., Castro-Mosquera M., Rodríguez-Arrizabalaga M., Debasa-Mouce M., Romaus-Sanjurjo D., Aramburu-Nuñez M., Iglesias-Rey R., Casas J., Lema I., Castillo J. (2025). Serum Levels of Autotaxin Reveal Its Role as a Novel Biomarker of Migraine. Headache.

[24] Bhattarai S., Sharma S., Ara H., Subedi U., Sun G., Li C., Shenuarin Bhuiyan M., Kevil C., Armstrong W.P., Minvielle M.T. (2021). Disrupted Blood-Brain Barrier and Mitochondrial Impairment by Autotaxin-Lysophosphatidic Acid Axis in Postischemic Stroke. J. Am. Heart Assoc..

[25] Perrakis A., Moolenaar W.H. (2014). Autotaxin: Structure-Function and Signaling. J. Lipid Res..

[26] Aoki J. (2004). Mechanisms of Lysophosphatidic Acid Production. Semin. Cell Dev. Biol..

[27] Aoki J., Inoue A., Okudaira S. (2008). Two Pathways for Lysophosphatidic Acid Production. Biochim. Biophys. Acta Mol. Cell Biol. Lipids.

[28] Stefan C., Jansen S., Bollen M. (2005). NPP-Type Ectophosphodiesterases: Unity in Diversity. Trends Biochem. Sci..

[29] Jansen S., Andries M., Vekemans K., Vanbilloen H., Verbruggen A., Bollen M. (2009). Rapid Clearance of the Circulating Metastatic Factor Autotaxin by the Scavenger Receptors of Liver Sinusoidal Endothelial Cells. Cancer Lett..

[30] Yung Y.C., Stoddard N.C., Chun J. (2014). LPA Receptor Signaling: Pharmacology, Physiology, and Pathophysiology. J. Lipid Res..

[31] Eichholtz T., Jalink K., Fahrenfort I., Moolenaar W.H. (1993). The Bioactive Phospholipid Lysophosphatidic Acid Is Released from Activated Platelets. Biochem. J..

[32] Ishii I., Fukushima N., Ye X., Chun J. (2004). Lysophospholipid Receptors: Signaling and Biology. Annu. Rev. Biochem..

[33] Hashimoto S., Mikami S., Sugino H., Yoshikawa A., Hashimoto A., Onodera Y., Furukawa S., Handa H., Oikawa T., Okada Y. (2016). Lysophosphatidic Acid Activates Arf6 to Promote the Mesenchymal Malignancy of Renal Cancer. Nat. Commun..

[34] Leblanc R., Peyruchaud O. (2015). New Insights into the Autotaxin/LPA Axis in Cancer Development and Metastasis. Exp. Cell Res..

[35] Lizalek J., McKenna T., Huegel K., Marsh S., Carolan A., Kobliska A., Heying E., Gardner N., Miller G., Kotecki A. (2015). Lysophosphatidic Acid Stimulates Urokinase Receptor (UPAR/CD87) in Ovarian Epithelial Cancer Cells. Anticancer Res..

[36] Yu X., Zhang Y., Chen H. (2016). LPA Receptor 1 Mediates LPA-Induced Ovarian Cancer Metastasis: An in Vitro and in Vivo Study. BMC Cancer.

[37] Hwang H., Kim E.-K., Park J., Suh P.-G., Cho Y.-K. (2014). RhoA and Rac1 Play Independent Roles in Lysophosphatidic Acid-Induced Ovarian Cancer Chemotaxis. Integr. Biol..

[38] Li Y.-Y., Zhang W.-C., Zhang J.-L., Zheng C.-J., Zhu H., Yu H.-M., Fan L.-M. (2015). Plasma Levels of Lysophosphatidic Acid in Ovarian Cancer versus Controls: A Meta-Analysis. Lipids Health Dis..

[39] Barbayianni E., Kaffe E., Aidinis V., Kokotos G. (2015). Autotaxin, a Secreted Lysophospholipase D, as a Promising Therapeutic Target in Chronic Inflammation and Cancer. Prog. Lipid Res..

[40] Kishi Y., Okudaira S., Tanaka M., Hama K., Shida D., Kitayama J., Yamori T., Aoki J., Fujimaki T., Arai H. (2006). Autotaxin Is Overexpressed in Glioblastoma Multiforme and Contributes to Cell Motility of Glioblastoma by Converting Lysophosphatidylcholine TO Lysophosphatidic Acid. J. Biol. Chem..

[41] Tabuchi S. (2015). The Autotaxin-Lysophosphatidic Acid–Lysophosphatidic Acid Receptor Cascade: Proposal of a Novel Potential Therapeutic Target for Treating Glioblastoma Multiforme. Lipids Health Dis..

[42] Allanore Y., Distler O., Jagerschmidt A., Illiano S., Ledein L., Boitier E., Agueusop I., Denton C.P., Khanna D. (2018). Lysophosphatidic Acid Receptor 1 Antagonist SAR100842 for Patients with Diffuse Cutaneous Systemic Sclerosis. Arthritis Rheumatol..

[43] Magkrioti C., Galaris A., Kanellopoulou P., Stylianaki E.A., Kaffe E., Aidinis V. (2019). Autotaxin and Chronic Inflammatory Diseases. J. Autoimmun..

[44] Oikonomou N., Mouratis M.-A., Tzouvelekis A., Kaffe E., Valavanis C., Vilaras G., Karameris A., Prestwich G.D., Bouros D., Aidinis V. (2012). Pulmonary Autotaxin Expression Contributes to the Pathogenesis of Pulmonary Fibrosis. Am. J. Respir. Cell Mol. Biol..

[45] Tager A.M. (2008). The Lysophosphatidic Acid Receptor LPA1 Links Pulmonary Fibrosis to Lung Injury by Mediating Fibroblast Recruitment and Vascular Leak. Nat. Med..

[46] Xiang H., Lu Y., Shao M., Wu T. (2020). Lysophosphatidic Acid Receptors: Biochemical and Clinical Implications in Different Diseases. J. Cancer.

[47] Ueda H. (2011). Lysophosphatidic Acid as the Initiator of Neuropathic Pain. Biol. Pharm. Bull..

[48] Ueda H. (2017). Lysophosphatidic Acid Signaling Is the Definitive Mechanism Underlying Neuropathic Pain. Pain.

[49] Roberts C., Winter P., Shilliam C.S., Hughes Z.A., Langmead C., Maycox P.R., Dawson L.A. (2005). Neurochemical Changes in LPA1 Receptor Deficient Mice—A Putative Model of Schizophrenia. Neurochem. Res..

[50] Stoddard N.C., Chun J. (2015). Promising Pharmacological Directions in the World of Lysophosphatidic Acid Signaling. Biomol. Ther..

[51] Choi J.W., Herr D.R., Noguchi K., Yung Y.C., Lee C.-W., Mutoh T., Lin M.-E., Teo S.T., Park K.E., Mosley A.N. (2010). LPA Receptors: Subtypes and Biological Actions. Annu. Rev. Pharmacol. Toxicol..

[52] Kihara Y., Maceyka M., Spiegel S., Chun J. (2014). Lysophospholipid Receptor Nomenclature Review: IUPHAR Review 8. Br. J. Pharmacol..

[53] Chun J., Hla T., Lynch K.R., Spiegel S., Moolenaar W.H. (2010). International Union of Basic and Clinical Pharmacology. LXXVIII. Lysophospholipid Receptor Nomenclature: TABLE 1. Pharmacol. Rev..

[54] Yanagida K., Kurikawa Y., Shimizu T., Ishii S. (2013). Current Progress in Non-Edg Family LPA Receptor Research. Biochim. Biophys. Acta (BBA)-Mol. Cell Biol. Lipids.

[55] Choi J.W., Chun J. (2013). Lysophospholipids and Their Receptors in the Central Nervous System. Biochim. Biophys. Acta (BBA)-Mol. Cell Biol. Lipids.

[56] Estivill-Torrús G., Santín L.J., Pedraza C., Castilla-Ortega E., Rodríguez De Fonseca F. (2013). Role of Lysophosphatidic Acid (LPA) in Behavioral Processes: Implications for Psychiatric Disorders. Lysophospholipid Receptors: Signaling and Biochemistry.

[57] Hu H.B., Song Z.Q., Song G.P., Li S., Tu H.Q., Wu M., Zhang Y.C., Yuan J.F., Li T.T., Li P.Y. (2021). LPA Signaling Acts as a Cell-Extrinsic Mechanism to Initiate Cilia Disassembly and Promote Neurogenesis. Nat. Commun..

[58] Dubin A.E., Herr D.R., Chun J. (2010). Diversity of Lysophosphatidic Acid Receptor-Mediated Intracellular Calcium Signaling in Early Cortical Neurogenesis. J. Neurosci..

[59] Suckau O., Gross I., Schrötter S., Yang F., Luo J., Wree A., Chun J., Baska D., Baumgart J., Kano K. (2019). LPA1, LPA2, LPA4 and LPA6 Receptor Expression during Mouse Brain Development. Dev. Dyn..

[60] Fujiwara Y., Sebök A., Meakin S., Kobayashi T., Murakami-Murofushi K., Tigyi G. (2003). Cyclic Phosphatidic Acid Elicits Neurotrophin-like Actions in Embryonic Hippocampal Neurons. J. Neurochem..

[61] Roza C., Campos-Sandoval J.A., Gómez-García M.C., Peñalver A., Márquez J. (2019). Lysophosphatidic Acid and Glutamatergic Transmission. Front. Mol. Neurosci..

[62] Hao Y., Guo M., Feng Y., Dong Q., Cui M. (2020). Lysophospholipids and Their G-Coupled Protein Signaling in Alzheimer’s Disease: From Physiological Performance to Pathological Impairment. Front. Mol. Neurosci..

[63] Plastira I., Bernhart E., Goeritzer M., DeVaney T., Reicher H., Hammer A., Lohberger B., Wintersperger A., Zucol B., Graier W.F. (2017). Lysophosphatidic Acid via LPA-Receptor 5/Protein Kinase D-Dependent Pathways Induces a Motile and pro-Inflammatory Microglial Phenotype. J. Neuroinflammation.

[64] Dedoni S., Avdoshina V., Olianas M.C., Onali P. (2025). Role of Lysophosphatidic Acid in Neurological Diseases: From Pathophysiology to Therapeutic Implications. Front. Biosci..

[65] Pereira-Castelo G., Bengoetxea de Tena I., Martínez-Gardeazabal J., Moreno-Rodríguez M., de San Román E.G., Manuel I., Rodríguez-Puertas R. (2024). Neurolipid Systems: A New Target for the Treatment of Dementia. Basic Clin. Pharmacol. Toxicol..

[66] Jose A., Fernando J.J., Kienesberger P.C. (2024). Lysophosphatidic Acid Metabolism and Signaling in Heart Disease. Can. J. Physiol. Pharmacol..

[67] Chattopadhyay A., Reddy S.T., Fogelman A.M. (2023). The Multiple Roles of Lysophosphatidic Acid in Vascular Disease and Atherosclerosis. Curr. Opin. Infect. Dis..

[68] Kume H., Harigane R., Rikimaru M. (2024). Involvement of Lysophospholipids in Pulmonary Vascular Functions and Diseases. Biomedicines.

[69] Yun C.C., Han Y., McConnell B. (2024). Lysophosphatidic Acid Signaling in the Gastrointestinal System. Cell Mol. Gastroenterol. Hepatol..

[70] Laface C., Ricci A.D., Vallarelli S., Ostuni C., Rizzo A., Ambrogio F., Centonze M., Schirizzi A., De Leonardis G., D’Alessandro R. (2024). Autotaxin-Lysophosphatidate Axis: Promoter of Cancer Development and Possible Therapeutic Implications. Int. J. Mol. Sci..

[71] Musazzi L., Di Daniel E., Maycox P., Racagni G., Popoli M. (2011). Abnormalities in α/β-CaMKII and Related Mechanisms Suggest Synaptic Dysfunction in Hippocampus of LPA1 Receptor Knockout Mice. Int. J. Neuropsychopharmacol..

[72] Zhang J., Li Y., Wang C., Wang Y., Zhang Y., Huang L., Zhang Z. (2020). Lysophosphatidic Acid Induces Apoptosis of PC12 Cells Through LPA1 Receptor/LPA2 Receptor/MAPK Signaling Pathway. Front. Mol. Neurosci..

[73] Lee C.-H., Sapkota A., Gaire B.P., Choi J.W. (2020). NLRP3 Inflammasome Activation Is Involved in LPA1-Mediated Brain Injury after Transient Focal Cerebral Ischemia. Int. J. Mol. Sci..

[74] Gaire B.P., Sapkota A., Song M.-R., Choi J.W. (2019). Lysophosphatidic Acid Receptor 1 (LPA1) Plays Critical Roles in Microglial Activation and Brain Damage after Transient Focal Cerebral Ischemia. J. Neuroinflammation.

[75] Halder S.K., Yano R., Chun J., Ueda H. (2013). Involvement of LPA1 Receptor Signaling in Cerebral Ischemia-Induced Neuropathic Pain. Neuroscience.

[76] Ueda H. (2019). Systems Pathology of Neuropathic Pain and Fibromyalgia. Biol. Pharm. Bull..

[77] Gao L., Shi H., Sherchan P., Tang H., Peng L., Xie S., Liu R., Hu X., Tang J., Xia Y. (2021). Inhibition of Lysophosphatidic Acid Receptor 1 Attenuates Neuroinflammation via PGE2/EP2/NOX2 Signalling and Improves the Outcome of Intracerebral Haemorrhage in Mice. Brain Behav. Immun..

[78] Lummis N.C., Sánchez-Pavón P., Kennedy G., Frantz A.J., Kihara Y., Blaho V.A., Chun J. (2019). LPA_1/3_ Overactivation Induces Neonatal Posthemorrhagic Hydrocephalus through Ependymal Loss and Ciliary Dysfunction. Sci. Adv..

[79] Amaral R.F.D., Geraldo L.H.M., Einicker-Lamas M., Spohr T.C.L.d.S.e., Mendes F., Lima F.R.S. (2021). Microglial Lysophosphatidic Acid Promotes Glioblastoma Proliferation and Migration via LPA_1_ Receptor. J. Neurochem..

[80] Valdés-Rives S.A., Arcos-Montoya D., de la Fuente-Granada M., Zamora-Sánchez C.J., Arias-Romero L.E., Villamar-Cruz O., Camacho-Arroyo I., Pérez-Tapia S.M., González-Arenas A. (2021). LPA1 Receptor Promotes Progesterone Receptor Phosphorylation through PKCα in Human Glioblastoma Cells. Cells.

[81] Fransson J., Gómez-Conde A.I., Romero-Imbroda J., Fernández O., Leyva L., de Fonseca F.R., Chun J., Louapre C., Van-Evercooren A.B., Zujovic V. (2021). Activation of Macrophages by Lysophosphatidic Acid through the Lysophosphatidic Acid Receptor 1 as a Novel Mechanism in Multiple Sclerosis Pathogenesis. Mol. Neurobiol..

[82] Gento-Caro Á., Vilches-Herrando E., García-Morales V., Portillo F., Rodríguez-Bey G., González-Forero D., Moreno-López B. (2021). Interfering with Lysophosphatidic Acid Receptor Edg2/Lpa_1_ Signalling Slows down Disease Progression in *SOD1-G93A* Transgenic Mice. Neuropathol. Appl. Neurobiol..

[83] Nam S.M., Choi J.H., Choi S.-H., Cho H.-J., Cho Y.-J., Rhim H., Kim H.-C., Cho I.-H., Kim D.-G., Nah S.-Y. (2021). Ginseng Gintonin Alleviates Neurological Symptoms in the G93A-SOD1 Transgenic Mouse Model of Amyotrophic Lateral Sclerosis through Lysophosphatidic Acid 1 Receptor. J. Ginseng Res..

[84] Valcárcel-Martín R., Martín-Suárez S., Muro-García T., Pastor-Alonso O., Rodríguez de Fonseca F., Estivill-Torrús G., Encinas J.M. (2020). Lysophosphatidic Acid Receptor 1 Specifically Labels Seizure-Induced Hippocampal Reactive Neural Stem Cells and Regulates Their Division. Front. Neurosci..

[85] Santos-Nogueira E., Lopez-Serrano C., Hernandez J., Lago N., Astudillo A.M., Balsinde J., Estivill-Torrus G., de Fonseca F.R., Chun J., Lopez-Vales R. (2015). Activation of Lysophosphatidic Acid Receptor Type 1 Contributes to Pathophysiology of Spinal Cord Injury. J. Neurosci..

[86] Hisaoka-Nakashima K., Yokoe T., Watanabe S., Nakamura Y., Kajitani N., Okada-Tsuchioka M., Takebayashi M., Nakata Y., Morioka N. (2021). Lysophosphatidic Acid Induces Thrombospondin-1 Production in Primary Cultured Rat Cortical Astrocytes. J. Neurochem..

[87] Olianas M.C., Dedoni S., Onali P. (2019). Inhibition of TNF-α-Induced Neuronal Apoptosis by Antidepressants Acting through the Lysophosphatidic Acid Receptor LPA1. Apoptosis.

[88] Rosell-Valle C., Martínez-Losa M., Matas-Rico E., Castilla-Ortega E., Zambrana-Infantes E., Gómez-Conde A.I., Sánchez-Salido L., Ladrón de Guevara-Miranda D., Pedraza C., Serrano-Castro P.J. (2021). GABAergic Deficits in Absence of LPA1 Receptor, Associated Anxiety-like and Coping Behaviors, and Amelioration by Interneuron Precursor Transplants into the Dorsal Hippocampus. Brain Struct. Funct..

[89] Moreno-Fernández R.D., Rosell-Valle C., Bacq A., Zanoletti O., Cifuentes M., Pérez-Martín M., Gavito A.L., García-Fernández M.I., Estivill-Torrús G., Rodríguez de Fonseca F. (2020). LPA1 Receptor and Chronic Stress: Effects on Behaviour and the Genes Involved in the Hippocampal Excitatory/Inhibitory Balance. Neuropharmacology.

[90] Tabbai S., Moreno-Fernández R.D., Zambrana-Infantes E., Nieto-Quero A., Chun J., García-Fernández M., Estivill-Torrús G., Rodríguez de Fonseca F., Santín L.J., Oliveira T.G. (2019). Effects of the LPA1 Receptor Deficiency and Stress on the Hippocampal LPA Species in Mice. Front. Mol. Neurosci..

[91] Puigdomenech-Poch M., Martínez-Muriana A., Andrés-Benito P., Ferrer I., Chun J., López-Vales R. (2021). Dual Role of Lysophosphatidic Acid Receptor 2 (LPA2) in Amyotrophic Lateral Sclerosis. Front. Cell Neurosci..

[92] López-Serrano C., Santos-Nogueira E., Francos-Quijorna I., Coll-Miró M., Chun J., López-Vales R. (2019). Lysophosphatidic Acid Receptor Type 2 Activation Contributes to Secondary Damage after Spinal Cord Injury in Mice. Brain Behav. Immun..

[93] Yang Y., Xu J., Su Q., Wu Y., Li Q., Ma Z., Ding T. (2022). Lysophosphatidic Acid Induced Apoptosis, DNA Damage, and Oxidative Stress in Spinal Cord Neurons by Upregulating LPA4/LPA6 Receptors. Mediat. Inflamm..

[94] Sapkota A., Lee C.-H., Park S.J., Choi J.W. (2020). Lysophosphatidic Acid Receptor 5 Plays a Pathogenic Role in Brain Damage after Focal Cerebral Ischemia by Modulating Neuroinflammatory Responses. Cells.

[95] Lin M.-E., Rivera R.R., Chun J. (2012). Targeted Deletion of LPA5 Identifies Novel Roles for Lysophosphatidic Acid Signaling in Development of Neuropathic Pain. J. Biol. Chem..

[96] Tsukahara R., Yamamoto S., Yoshikawa K., Gotoh M., Tsukahara T., Neyama H., Ishii S., Akahoshi N., Yanagida K., Sumida H. (2018). LPA5 Signaling Is Involved in Multiple Sclerosis-Mediated Neuropathic Pain in the Cuprizone Mouse Model. J. Pharmacol. Sci..

[97] Joshi L., Plastira I., Bernhart E., Reicher H., Koshenov Z., Graier W.F., Vujic N., Kratky D., Rivera R., Chun J. (2022). Lysophosphatidic Acid Receptor 5 (LPA5) Knockout Ameliorates the Neuroinflammatory Response In Vivo and Modifies the Inflammatory and Metabolic Landscape of Primary Microglia In Vitro. Cells.

[98] Masago K., Kihara Y., Yanagida K., Hamano F., Nakagawa S., Niwa M., Shimizu T. (2018). Lysophosphatidic Acid Receptor, LPA6, Regulates Endothelial Blood-Brain Barrier Function: Implication of Hepatic Encephalopathy. Biochem. Biophys. Res. Commun..

[99] Spencer S.A., Suárez-Pozos E., Soto-Verdugo J., Wang H., Afshari F.S., Li G., Manam S., Yasuda D., Ortega A., Lister J.A. (2022). Lysophosphatidic Acid Signaling via LPA_6_: A Negative Modulator of Developmental Oligodendrocyte Maturation. J. Neurochem..

[100] Hecht J.H., Weiner J.A., Post S.R., Chun J. (1996). Ventricular Zone Gene-1 (Vzg-1) Encodes a Lysophosphatidic Acid Receptor Expressed in Neurogenic Regions of the Developing Cerebral Cortex. J. Cell Biol..

[101] Fukushima N., Kimura Y., Chun J. (1998). A Single Receptor Encoded by *Vzg-1*/*Lp*_A1_/*Edg-2* Couples to G Proteins and Mediates Multiple Cellular Responses to Lysophosphatidic Acid. Proc. Natl. Acad. Sci. USA.

[102] Liu S., Paknejad N., Zhu L., Kihara Y., Ray M., Chun J., Liu W., Hite R.K., Huang X.-Y. (2022). Differential Activation Mechanisms of Lipid GPCRs by Lysophosphatidic Acid and Sphingosine 1-Phosphate. Nat. Commun..

[103] Ray M., Nagai K., Kihara Y., Kussrow A., Kammer M.N., Frantz A., Bornhop D.J., Chun J. (2020). Unlabeled Lysophosphatidic Acid Receptor Binding in Free Solution as Determined by a Compensated Interferometric Reader. J. Lipid Res..

[104] Birgbauer E. (2020). Lysophosphatidic Acid Signalling in Nervous System Development and Function. Neuromol. Med..

[105] Yanagida K., Masago K., Nakanishi H., Kihara Y., Hamano F., Tajima Y., Taguchi R., Shimizu T., Ishii S. (2009). Identification and Characterization of a Novel Lysophosphatidic Acid Receptor, P2y5/LPA6. J. Biol. Chem..

[106] Contos J.J.A., Ishii I., Chun J. (2000). Lysophosphatidic Acid Receptors. Mol. Pharmacol..

[107] Weiner J.A., Hecht J.H., Chun J. (1998). Lysophosphatidic Acid Receptor Gene Vzg-1/Lp(A)1/Edg-2 Is Expressed by Mature Oligodendrocytes during Myelination in the Postnatal Murine Brain. J. Comp. Neurol..

[108] Allard J., Barrón S., Diaz J., Lubetzki C., Zalc B., Schwartz J.C., Sokoloff P. (1998). A Rat G Protein-Coupled Receptor Selectively Expressed in Myelin-Forming Cells. Eur. J. Neurosci..

[109] Fukushima N., Weiner J.A., Chun J. (2000). Lysophosphatidic Acid (LPA) Is a Novel Extracellular Regulator of Cortical Neuroblast Morphology. Dev. Biol..

[110] Kingsbury M.A., Rehen S.K., Contos J.J.A., Higgins C.M., Chun J. (2003). Non-Proliferative Effects of Lysophosphatidic Acid Enhance Cortical Growth and Folding. Nat. Neurosci..

[111] Estivill-Torrús G., Llebrez-Zayas P., Matas-Rico E., Santín L., Pedraza C., de Diego I., del Arco I., Fernández-Llebrez P., Chun J., de Fonseca F.R. (2008). Absence of LPA1 Signaling Results in Defective Cortical Development. Cereb. Cortex.

[112] Fukushima N., Weiner J.A., Kaushal D., Contos J.J.A., Rehen S.K., Kingsbury M.A., Kim K.Y., Chun J. (2002). Lysophosphatidic Acid Influences the Morphology and Motility of Young, Postmitotic Cortical Neurons. Mol. Cell. Neurosci..

[113] Weiner J.A., Fukushima N., Contos J.J.A., Scherer S.S., Chun J. (2001). Regulation of Schwann Cell Morphology and Adhesion by Receptor-Mediated Lysophosphatidic Acid Signaling. J. Neurosci..

[114] Matas-Rico E., García-Diaz B., Llebrez-Zayas P., López-Barroso D., Santín L., Pedraza C., Smith-Fernández A., Fernández-Llebrez P., Tellez T., Redondo M. (2008). Deletion of Lysophosphatidic Acid Receptor LPA1 Reduces Neurogenesis in the Mouse Dentate Gyrus. Mol. Cell. Neurosci..

[115] Yang A. (2002). In Vivo Roles of Lysophospholipid Receptors Revealed by Gene Targeting Studies in Mice. Biochim. Biophys. Acta (BBA)-Mol. Cell Biol. Lipids.

[116] Contos J.J.A., Fukushima N., Weiner J.A., Kaushal D., Chun J. (2000). Requirement for the *Lp*_A1_ Lysophosphatidic Acid Receptor Gene in Normal Suckling Behavior. Proc. Natl. Acad. Sci. USA.

[117] Rivera R., Williams N.A., Kennedy G.G., Sánchez-Pavón P., Chun J. (2021). Generation of an Lpar1-EGFP Fusion Knock-in Transgenic Mouse Line. Cell Biochem. Biophys..

[118] Kajitani N., Okada-Tsuchioka M., Kano K., Omori W., Boku S., Aoki J., Takebayashi M. (2020). Differential Anatomical and Cellular Expression of Lysophosphatidic Acid Receptor 1 in Adult Mouse Brain. Biochem. Biophys. Res. Commun..

[119] Uchida H., Nagai J., Ueda H. (2014). Lysophosphatidic Acid and Its Receptors LPA1 and LPA3 Mediate Paclitaxel-Induced Neuropathic Pain in Mice. Mol. Pain.

[120] Ueda H., Neyama H., Matsushita Y. (2020). Lysophosphatidic Acid Receptor 1- and 3-Mediated Hyperalgesia and Hypoalgesia in Diabetic Neuropathic Pain Models in Mice. Cells.

[121] Ueda H., Neyama H., Sasaki K., Miyama C., Iwamoto R. (2018). Lysophosphatidic Acid LPA1 and LPA3 Receptors Play Roles in the Maintenance of Late Tissue Plasminogen Activator-Induced Central Poststroke Pain in Mice. Neurobiol. Pain.

[122] Contos J.J.A., Chun J. (2000). Genomic Characterization of the Lysophosphatidic Acid Receptor Gene, LpA2/Edg4, and Identification of a Frameshift Mutation in a Previously Characterized CDNA. Genomics.

[123] Uenaka M., Uyeda A., Nakahara T., Muramatsu R. (2022). LPA2 Promotes Neuronal Differentiation and Neurite Formation in Neocortical Development. Biochem. Biophys. Res. Commun..

[124] Ohuchi H., Hamada A., Matsuda H., Takagi A., Tanaka M., Aoki J., Arai H., Noji S. (2008). Expression Patterns of the Lysophospholipid Receptor Genes during Mouse Early Development. Dev. Dyn..

[125] Contos J.J.A., Ishii I., Fukushima N., Kingsbury M.A., Ye X., Kawamura S., Brown J.H., Chun J. (2002). Characterization of Lpa(2) (Edg4) and Lpa(1)/Lpa(2) (Edg2/Edg4) Lysophosphatidic Acid Receptor Knockout Mice: Signaling Deficits without Obvious Phenotypic Abnormality Attributable to Lpa(2). Mol. Cell Biol..

[126] Frugier T., Crombie D., Conquest A., Tjhong F., Taylor C., Kulkarni T., McLean C., Pébay A. (2011). Modulation of LPA Receptor Expression in the Human Brain Following Neurotrauma. Cell Mol. Neurobiol..

[127] Goldshmit Y., Munro K., Leong S.Y., Pébay A., Turnley A.M. (2010). LPA Receptor Expression in the Central Nervous System in Health and Following Injury. Cell Tissue Res..

[128] Savitz S.I., Dhallu M.S., Malhotra S., Mammis A., Ocava L.C., Rosenbaum P.S., Rosenbaum D.M. (2006). EDG Receptors as a Potential Therapeutic Target in Retinal Ischemia-Reperfusion Injury. Brain Res..

[129] Ye X., Hama K., Contos J.J.A., Anliker B., Inoue A., Skinner M.K., Suzuki H., Amano T., Kennedy G., Arai H. (2005). LPA3-Mediated Lysophosphatidic Acid Signalling in Embryo Implantation and Spacing. Nature.

[130] Zhao P., Yun Q., Li A., Li R., Yan Y., Wang Y., Sun H., Damirin A. (2022). LPA3 Is a Precise Therapeutic Target and Potential Biomarker for Ovarian Cancer. Med. Oncol..

[131] Furuta D., Yamane M., Tsujiuchi T., Moriyama R., Fukushima N. (2012). Lysophosphatidic Acid Induces Neurite Branch Formation through LPA3. Mol. Cell. Neurosci..

[132] Solís K.H., Romero-Ávila M.T., Guzmán-Silva A., García-Sáinz J.A. (2021). The LPA3 Receptor: Regulation and Activation of Signaling Pathways. Int. J. Mol. Sci..

[133] Acton S., O’Donnell M.M., Periyasamy K., Dixit B., Eishingdrelo H., Hill C., Paul Ross R., Chesnel L. (2024). LPA3 Agonist-Producing Bacillus Velezensis ADS024 Is Efficacious in Multiple Neuroinflammatory Disease Models. Brain Behav. Immun..

[134] Bandoh K., Aoki J., Taira A., Tsujimoto M., Arai H., Inoue K. (2000). Lysophosphatidic Acid (LPA) Receptors of the EDG Family Are Differentially Activated by LPA Species. FEBS Lett..

[135] Noguchi K., Ishii S., Shimizu T. (2003). Identification of P2y9/GPR23 as a Novel G Protein-Coupled Receptor for Lysophosphatidic Acid, Structurally Distant from the Edg Family. J. Biol. Chem..

[136] Yanagida K., Ishii S., Hamano F., Noguchi K., Shimizu T. (2007). LPA4/P2y9/GPR23 Mediates Rho-Dependent Morphological Changes in a Rat Neuronal Cell Line. J. Biol. Chem..

[137] Lee C.W., Rivera R., Dubin A.E., Chun J. (2007). LPA4/GPR23 Is a Lysophosphatidic Acid (LPA) Receptor Utilizing Gs-, Gq/Gi-Mediated Calcium Signaling and G12/13-Mediated Rho Activation. J. Biol. Chem..

[138] Lee Z., Cheng C.-T., Zhang H., Subler M.A., Wu J., Mukherjee A., Windle J.J., Chen C.-K., Fang X. (2008). Role of LPA_4_ /P2y9/GPR23 in Negative Regulation of Cell Motility. Mol. Biol. Cell.

[139] Kurabayashi N., Tanaka A., Nguyen M.D., Sanada K. (2018). The LPA-LPA4 Axis Is Required for Establishment of Bipolar Morphology and Radial Migration of Newborn Cortical Neurons. Development.

[140] Lee C.W., Rivera R., Gardell S., Dubin A.E., Chun J. (2006). GPR92 as a New G12/13- and Gq-Coupled Lysophosphatidic Acid Receptor That Increases CAMP, LPA5. J. Biol. Chem..

[141] Callaerts-Vegh Z., Leo S., Vermaercke B., Meert T., D’Hooge R. (2012). LPA_5_ Receptor Plays a Role in Pain Sensitivity, Emotional Exploration and Reversal Learning. Genes. Brain Behav..

[142] Murai N., Hiyama H., Kiso T., Sekizawa T., Watabiki T., Oka H., Aoki T. (2017). Analgesic Effects of Novel Lysophosphatidic Acid Receptor 5 Antagonist AS2717638 in Rodents. Neuropharmacology.

[143] Plastira I., Bernhart E., Goeritzer M., Reicher H., Kumble V.B., Kogelnik N., Wintersperger A., Hammer A., Schlager S., Jandl K. (2016). 1-Oleyl-Lysophosphatidic Acid (LPA) Promotes Polarization of BV-2 and Primary Murine Microglia towards an M1-like Phenotype. J. Neuroinflamm..

[144] Gouras G.K., Olsson T.T., Hansson O. (2015). β-Amyloid Peptides and Amyloid Plaques in Alzheimer’s Disease. Neurotherapeutics.

[145] Mucke L., Selkoe D.J. (2012). Neurotoxicity of Amyloid -Protein: Synaptic and Network Dysfunction. Cold Spring Harb. Perspect. Med..

[146] Gao Y., Tan L., Yu J.-T., Tan L. (2018). Tau in Alzheimer’s Disease: Mechanisms and Therapeutic Strategies. Curr. Alzheimer Res..

[147] Cheignon C., Tomas M., Bonnefont-Rousselot D., Faller P., Hureau C., Collin F. (2018). Oxidative Stress and the Amyloid Beta Peptide in Alzheimer’s Disease. Redox Biol..

[148] Bassett C.N., Neely M.D., Sidell K.R., Markesbery W.R., Switt L.L., Montine T.J. (1999). Cerebrospinal Fluid Lipoproteins Are More Vulnerable to Oxidation in Alzheimer’s Disease and Are Neurotoxic When Oxidized Ex Vivo. Lipids.

[149] Trpkovic A., Resanovic I., Stanimirovic J., Radak D., Mousa S.A., Cenic-Milosevic D., Jevremovic D., Isenovic E.R. (2015). Oxidized Low-Density Lipoprotein as a Biomarker of Cardiovascular Diseases. Crit. Rev. Clin. Lab. Sci..

[150] Shi J., Dong Y., Cui M.-Z., Xu X. (2013). Lysophosphatidic Acid Induces Increased BACE1 Expression and Aβ Formation. Biochim. Biophys. Acta (BBA)-Mol. Basis Dis..

[151] Du Y., Zhao Y., Li C., Zheng Q., Tian J., Li Z., Huang T.Y., Zhang W., Xu H. (2018). Inhibition of PKCδ Reduces Amyloid-β Levels and Reverses Alzheimer Disease Phenotypes. J. Exp. Med..

[152] Lahiri D., Ge Y.-W., Rogers J., Sambamurti K., Greig N., Maloney B. (2006). Taking Down the Unindicted Co-Conspirators of Amyloid β-Peptidemediated Neuronal Death: Shared Gene Regulation of BACE1 and APP Genes Interacting with CREB, Fe65 and YY1 Transcription Factors. Curr. Alzheimer Res..

[153] Vassar R., Bennett B.D., Babu-Khan S., Kahn S., Mendiaz E.A., Denis P., Teplow D.B., Ross S., Amarante P., Loeloff R. (1999). β-Secretase Cleavage of Alzheimer’s Amyloid Precursor Protein by the Transmembrane Aspartic Protease BACE. Science.

[154] Xu X. (2009). γ-Secretase Catalyzes Sequential Cleavages of the AβPP Transmembrane Domain. J. Alzheimer’s Dis..

[155] Hampel H., Vassar R., de Strooper B., Hardy J., Willem M., Singh N., Zhou J., Yan R., Vanmechelen E., de Vos A. (2021). The β-Secretase BACE1 in Alzheimer’s Disease. Biol. Psychiatry.

[156] Shobab L.A., Hsiung G.-Y.R., Feldman H.H. (2005). Cholesterol in Alzheimer’s Disease. Lancet Neurol..

[157] Fantini J., Garmy N., Mahfoud R., Yahi N. (2002). Lipid Rafts: Structure, Function and Role in HIV, Alzheimer’s and Prion Diseases. Expert Rev. Mol. Med..

[158] Hicks D.A., Nalivaeva N.N., Turner A.J. (2012). Lipid Rafts and Alzheimer’s Disease: Protein-Lipid Interactions and Perturbation of Signaling. Front. Physiol..

[159] Dias I.H.K., Mistry J., Fell S., Reis A., Spickett C.M., Polidori M.C., Lip G.Y.H., Griffiths H.R. (2014). Oxidized LDL Lipids Increase β-Amyloid Production by SH-SY5Y Cells through Glutathione Depletion and Lipid Raft Formation. Free Radic. Biol. Med..

[160] Wang Y., Qiao M., Mieyal J.J., Asmis L.M., Asmis R. (2006). Molecular Mechanism of Glutathione-Mediated Protection from Oxidized Low-Density Lipoprotein-Induced Cell Injury in Human Macrophages: Role of Glutathione Reductase and Glutaredoxin. Free Radic. Biol. Med..

[161] Weingarten M.D., Lockwood A.H., Hwo S.Y., Kirschner M.W. (1975). A Protein Factor Essential for Microtubule Assembly. Proc. Natl. Acad. Sci. USA.

[162] Chaudhary N., Nagaraj R. (2012). Tau Fibrillogenesis. Subcell. Biochem..

[163] Sun Y., Kim N.-H., Yang H., Kim S.-H., Huh S.-O. (2011). Lysophosphatidic Acid Induces Neurite Retraction in Differentiated Neuroblastoma Cells via GSK-3β Activation. Mol. Cells.

[164] Sayas C. (2002). Regulation of Neuronal Cytoskeleton by Lysophosphatidic Acid: Role of GSK-3. Biochim. Biophys. Acta (BBA)-Mol. Cell Biol. Lipids.

[165] Takahashi M., Tomizawa K., Kato R., Sato K., Uchida T., Fujita S.C., Imahori K. (1994). Localization and Developmental Changes of τ Protein Kinase I/Glycogen Synthase Kinase-3β in Rat Brain. J. Neurochem..

[166] Lauretti E., Dincer O., Praticò D. (2020). Glycogen Synthase Kinase-3 Signaling in Alzheimer’s Disease. Biochim. Biophys. Acta (BBA)-Mol. Cell Res..

[167] Kim J.-G., Kim M.-J., Choi W.-J., Moon M.-Y., Kim H.-J., Lee J.-Y., Kim J., Kim S.-C., Kang S.G., Seo G.-Y. (2017). Wnt3A Induces GSK-3β Phosphorylation and β-Catenin Accumulation Through RhoA/ROCK. J. Cell. Physiol..

[168] Amano M., Kaneko T., Maeda A., Nakayama M., Ito M., Yamauchi T., Goto H., Fukata Y., Oshiro N., Shinohara A. (2003). Identification of Tau and MAP2 as Novel Substrates of Rho-Kinase and Myosin Phosphatase. J. Neurochem..

[169] Ebeid M.A., Habib M.Z., Mohamed A.M., el Faramawy Y., Saad S.S.T., El-Kharashi O.A., el Magdoub H.M., Abd-Alkhalek H.A., Aboul-Fotouh S., Abdel-Tawab A.M. (2021). Cognitive Effects of the GSK-3 Inhibitor “Lithium” in LPS/Chronic Mild Stress Rat Model of Depression: Hippocampal and Cortical Neuroinflammation and Tauopathy. Neurotoxicology.

[170] Sayas C.L., Moreno-Flores M.T., Avila J., Wandosell F. (1999). The Neurite Retraction Induced by Lysophosphatidic Acid Increases Alzheimer’s Disease-like Tau Phosphorylation. J. Biol. Chem..

[171] Munoz L., Ammit A.J. (2010). Targeting P38 MAPK Pathway for the Treatment of Alzheimer’s Disease. Neuropharmacology.

[172] Maldonado H., Ramírez E., Utreras E., Pando M.E., Kettlun A.M., Chiong M., Kulkarni A.B., Collados L., Puente J., Cartier L. (2011). Inhibition of Cyclin-Dependent Kinase 5 but Not of Glycogen Synthase Kinase 3-β Prevents Neurite Retraction and Tau Hyperphosphorylation Caused by Secretable Products of Human T-Cell Leukemia Virus Type I-Infected Lymphocytes. J. Neurosci. Res..

[173] Ahmad S., Orellana A., Kohler I., Frölich L., de Rojas I., Gil S., Boada M., Hernández I., Hausner L., Bakker M.H.M. (2020). Association of Lysophosphatidic Acids with Cerebrospinal Fluid Biomarkers and Progression to Alzheimer’s Disease. Alzheimers Res. Ther..

[174] Santin L.J., Bilbao A., Pedraza C., Matas-Rico E., López-Barroso D., Castilla-Ortega E., Sánchez-López J., Riquelme R., Varela-Nieto I., de la Villa P. (2009). Behavioral Phenotype of MaLPA_1_-Null Mice: Increased Anxiety-like Behavior and Spatial Memory Deficits. Genes Brain Behav..

[175] Castilla-Ortega E., Hoyo-Becerra C., Pedraza C., Chun J., Rodríguez De Fonseca F., Estivill-Torrús G., Santín L.J. (2011). Aggravation of Chronic Stress Effects on Hippocampal Neurogenesis and Spatial Memory in LPA1 Receptor Knockout Mice. PLoS ONE.

[176] Li Y., Fan H., Sun J., Ni M., Zhang L., Chen C., Hong X., Fang F., Zhang W., Ma P. (2020). Circular RNA Expression Profile of Alzheimer’s Disease and Its Clinical Significance as Biomarkers for the Disease Risk and Progression. Int. J. Biochem. Cell Biol..

[177] Wu L., Du Q., Wu C. (2021). CircLPAR1/MiR-212-3p/ZNF217 Feedback Loop Promotes Amyloid β-Induced Neuronal Injury in Alzheimer’s Disease. Brain Res..

[178] González de San Román E., Llorente-Ovejero A., Martínez-Gardeazabal J., Moreno-Rodríguez M., Giménez-Llort L., Manuel I., Rodríguez-Puertas R. (2021). Modulation of Neurolipid Signaling and Specific Lipid Species in the Triple Transgenic Mouse Model of Alzheimer’s Disease. Int. J. Mol. Sci..

[179] Umemura K., Yamashita N., Yu X., Arima K., Asada T., Makifuchi T., Murayama S., Saito Y., Kanamaru K., Goto Y. (2006). Autotaxin Expression Is Enhanced in Frontal Cortex of Alzheimer-Type Dementia Patients. Neurosci. Lett..

[180] McLimans K.E., Willette A.A. (2017). Autotaxin Is Related to Metabolic Dysfunction and Predicts Alzheimer’s Disease Outcomes. J. Alzheimer’s Dis..

[181] Armstrong R.A. (2019). Risk Factors for Alzheimer’s Disease. Folia Neuropathol..

[182] Cash A., Theus M.H. (2020). Mechanisms of Blood–Brain Barrier Dysfunction in Traumatic Brain Injury. Int. J. Mol. Sci..

[183] Yu Y., Qin J., Liu M., Ruan Q., Li Y., Zhang Z. (2014). Role of Rho Kinase in Lysophosphatidic Acid-Induced Altering of Blood-Brain Barrier Permeability. Int. J. Mol. Med..

[184] Ladrón de Guevara-Miranda D., Moreno-Fernández R.D., Gil-Rodríguez S., Rosell-Valle C., Estivill-Torrús G., Serrano A., Pavón F.J., Rodríguez de Fonseca F., Santín L.J., Castilla-Ortega E. (2019). Lysophosphatidic Acid-induced Increase in Adult Hippocampal Neurogenesis Facilitates the Forgetting of Cocaine-contextual Memory. Addict. Biol..

[185] Jaroudi W., Garami J., Garrido S., Hornberger M., Keri S., Moustafa A.A. (2017). Factors Underlying Cognitive Decline in Old Age and Alzheimer’s Disease: The Role of the Hippocampus. Rev. Neurosci..

[186] Jiang G., Xu Y., Fujiwara Y., Tsukahara T., Tsukahara R., Gajewiak J., Tigyi G., Prestwich G.D. (2007). α-Substituted Phosphonate Analogues of Lysophosphatidic Acid (LPA) Selectively Inhibit Production and Action of LPA. ChemMedChem.

[187] Taleb S.J., Wei J., Mialki R.K., Dong S., Li Y., Zhao J., Zhao Y. (2021). A Blocking Peptide Stabilizes Lysophosphatidic Acid Receptor 1 and Promotes Lysophosphatidic Acid-induced Cellular Responses. J. Cell. Biochem..

[188] Gaire B.P., Sapkota A., Choi J.W. (2020). BMS-986020, a Specific LPA1 Antagonist, Provides Neuroprotection against Ischemic Stroke in Mice. Antioxidants.

[189] Kano K., Arima N., Ohgami M., Aoki J. (2008). LPA and Its Analogs-Attractive Tools for Elucidation of LPA Biology and Drug Development. Curr. Med. Chem..

[190] Holtsberg F.W., Steiner M.R., Keller J.N., Mark R.J., Mattson M.P., Steiner S.M. (1998). Lysophosphatidic Acid Induces Necrosis and Apoptosis in Hippocampal Neurons. J. Neurochem..

[191] Rosell-Valle C., Pedraza C., Manuel I., Moreno-Rodríguez M., Rodríguez-Puertas R., Castilla-Ortega E., Caramés J.M., Gómez Conde A.I., Zambrana-Infantes E., Ortega-Pinazo J. (2021). Chronic Central Modulation of LPA/LPA Receptors-Signaling Pathway in the Mouse Brain Regulates Cognition, Emotion, and Hippocampal Neurogenesis. Prog. Neuropsychopharmacol. Biol. Psychiatry.

[192] Olianas M.C., Dedoni S., Onali P. (2017). LPA_1_ Is a Key Mediator of Intracellular Signalling and Neuroprotection Triggered by Tetracyclic Antidepressants in Hippocampal Neurons. J. Neurochem..

[193] Zotova E., Bharambe V., Cheaveau M., Morgan W., Holmes C., Harris S., Neal J.W., Love S., Nicoll J.A.R., Boche D. (2013). Inflammatory Components in Human Alzheimer’s Disease and after Active Amyloid-Β42 Immunization. Brain.

[194] Sun Y.X., Minthon L., Wallmark A., Warkentin S., Blennow K., Janciauskiene S. (2003). Inflammatory Markers in Matched Plasma and Cerebrospinal Fluid from Patients with Alzheimer’s Disease. Dement. Geriatr. Cogn. Disord..

[195] Dani M., Wood M., Mizoguchi R., Fan Z., Walker Z., Morgan R., Hinz R., Biju M., Kuruvilla T., Brooks D.J. (2018). Microglial Activation Correlates in Vivo with Both Tau and Amyloid in Alzheimer’s Disease. Brain.

[196] Pereira C.F., Santos A.E., Moreira P.I., Pereira A.C., Sousa F.J., Cardoso S.M., Cruz M.T. (2019). Is Alzheimer’s Disease an Inflammasomopathy?. Ageing Res. Rev..

[197] Plastira I., Bernhart E., Joshi L., Koyani C.N., Strohmaier H., Reicher H., Malle E., Sattler W. (2020). MAPK Signaling Determines Lysophosphatidic Acid (LPA)-Induced Inflammation in Microglia. J. Neuroinflammation.

[198] Ninou I., Sevastou I., Magkrioti C., Kaffe E., Stamatakis G., Thivaios S., Panayotou G., Aoki J., Kollias G., Aidinis V. (2020). Genetic Deletion of Autotaxin from CD11b+ Cells Decreases the Severity of Experimental Autoimmune Encephalomyelitis. PLoS ONE.

[199] Herr D.R., Ong J.H.-J., Ong W.-Y. (2018). Potential Therapeutic Applications for Inhibitors of Autotaxin, a Bioactive Lipid-Producing Lysophospholipase D, in Disorders Affecting the Nervous System. ACS Chem. Neurosci..

[200] Maher T.M., van der Aar E.M., Van de Steen O., Allamassey L., Desrivot J., Dupont S., Fagard L., Ford P., Fieuw A., Wuyts W. (2018). Safety, Tolerability, Pharmacokinetics, and Pharmacodynamics of GLPG1690, a Novel Autotaxin Inhibitor, to Treat Idiopathic Pulmonary Fibrosis (FLORA): A Phase 2a Randomised Placebo-Controlled Trial. Lancet Respir. Med..

[201] Yang L., Shu P., Wu N., Hu M., Luo Z. (2023). Pharmacokinetics, Pharmacodynamics, Safety and Tolerability of FTP-198, a Novel, Selective Autotaxin Inhibitor, in Healthy Subjects: A Phase I Randomized Placebo-Controlled Trial. Eur. J. Pharm. Sci..

[202] Khanna D., Denton C.P., Furst D.E., Mayes M.D., Matucci-Cerinic M., Smith V., de Vries D., Ford P., Bauer Y., Randall M.J. (2023). A 24-Week, Phase IIa, Randomized, Double-Blind, Placebo-Controlled Study of Ziritaxestat in Early Diffuse Cutaneous Systemic Sclerosis. Arthritis Rheumatol..

[203] Maher T.M., Ford P., Brown K.K., Costabel U., Cottin V., Danoff S.K., Groenveld I., Helmer E., Jenkins R.G., Milner J. (2023). Ziritaxestat, a Novel Autotaxin Inhibitor, and Lung Function in Idiopathic Pulmonary Fibrosis: The ISABELA 1 and 2 Randomized Clinical Trials. JAMA.

[204] Ma B., Zhang L., Sun L., Xin Z., Kumaravel G., Marcotte D., Chodaparambil J.V., Wang Q., Wehr A., Jing J. (2021). Discovery of Potent Selective Nonzinc Binding Autotaxin Inhibitor BIO-32546. ACS Med. Chem. Lett..

[205] Eymery M.C., Nguyen K.-A., Basu S., Hausmann J., Tran-Nguyen V.-K., Seidel H.P., Gutierrez L., Boumendjel A., McCarthy A.A. (2024). Discovery of Potent Chromone-Based Autotaxin Inhibitors Inspired by Cannabinoids. Eur. J. Med. Chem..

[206] Clark J.M., Salgado-Polo F., Macdonald S.J.F., Barrett T.N., Perrakis A., Jamieson C. (2022). Structure-Based Design of a Novel Class of Autotaxin Inhibitors Based on Endogenous Allosteric Modulators. J. Med. Chem..

[207] Valentine W.J., Kiss G.N., Liu J., E S., Gotoh M., Murakami-Murofushi K., Pham T.C., Baker D.L., Parrill A.L., Lu X. (2010). (S)-FTY720-Vinylphosphonate, an Analogue of the Immunosuppressive Agent FTY720, Is a Pan-Antagonist of Sphingosine 1-Phosphate GPCR Signaling and Inhibits Autotaxin Activity. Cell. Signal..

[208] van Meeteren L.A., Brinkmann V., Saulnier-Blache J.S., Lynch K.R., Moolenaar W.H. (2008). Anticancer Activity of FTY720: Phosphorylated FTY720 Inhibits Autotaxin, a Metastasis-Enhancing and Angiogenic Lysophospholipase D. Cancer Lett..

[209] Szepanowski F., Derksen A., Steiner I., Meyer zu Hörste G., Daldrup T., Hartung H.P., Kieseier B.C.S. (2016). Fingolimod Promotes Peripheral Nerve Regeneration via Modulation of Lysophospholipid Signaling. J. Neuroinflammation.

[210] Roy S., Chakrabarti M., Dasgupta H., Mahale A., Tripathi S., Sharma V., Banerjee M., Kulkarni O.P. (2022). Inhibition of Autotaxin Ameliorates LPA-Mediated Neuroinflammation and Alleviates Neurological Dysfunction in Acute Hepatic Encephalopathy. ACS Chem. Neurosci..

[211] Nah S.-Y. (2012). Gintonin: A Novel Ginseng-Derived Ligand That Targets G Protein- Coupled Lysophosphatidic Acid Receptors. Curr. Drug Targets.

[212] Jakaria M., Azam S., Go E.-A., Uddin M.S., Jo S.-H., Choi D.-K. (2021). Biological Evidence of Gintonin Efficacy in Memory Disorders. Pharmacol. Res..

[213] Moon J., Choi S.-H., Shim J.-Y., Park H.-J., Oh M.-J., Kim M., Nah S.-Y. (2018). Gintonin Administration Is Safe and Potentially Beneficial in Cognitively Impaired Elderly. Alzheimer Dis. Assoc. Disord..

[214] Hwang S.H., Shin E.-J., Shin T.-J., Lee B.-H., Choi S.-H., Kang J., Kim H.-J., Kwon S.-H., Jang C.-G., Lee J.-H. (2012). Gintonin, a Ginseng-Derived Lysophosphatidic Acid Receptor Ligand, Attenuates Alzheimer’s Disease-Related Neuropathies: Involvement of Non-Amyloidogenic Processing. J. Alzheimer’s Dis..

[215] Cho Y.-J., Choi S.-H., Lee R.-M., Cho H.-S., Rhim H., Kim H.-C., Kim B.-J., Kim J.-H., Nah S.-Y. (2021). Protective Effects of Gintonin on Reactive Oxygen Species-Induced HT22 Cell Damages: Involvement of LPA1 Receptor-BDNF-AKT Signaling Pathway. Molecules.

[216] Kim D.-G., Kim H.-J., Choi S.-H., Nam S.M., Kim H.-C., Rhim H., Cho I.-H., Rhee M.H., Nah S.-Y. (2021). Gintonin Influences the Morphology and Motility of Adult Brain Neurons via LPA Receptors. J. Ginseng Res..

[217] Kim D.-G., Jang M., Choi S.-H., Kim H.-J., Jhun H., Kim H.-C., Rhim H., Cho I.-H., Nah S.-Y. (2018). Gintonin, a Ginseng-Derived Exogenous Lysophosphatidic Acid Receptor Ligand, Enhances Blood-Brain Barrier Permeability and Brain Delivery. Int. J. Biol. Macromol..

[218] Dooley M., Lamb H.M. (2000). Donepezil. Drugs Aging.

[219] Choi S.-H., Lee N.-E., Cho H.-J., Lee R.M., Rhim H., Kim H.-C., Han M., Lee E.-H., Park J., Kim J.N. (2021). Gintonin Facilitates Brain Delivery of Donepezil, a Therapeutic Drug for Alzheimer Disease, through Lysophosphatidic Acid 1/3 and Vascular Endothelial Growth Factor Receptors. J. Ginseng Res..

[220] Crack P.J., Zhang M., Morganti-Kossmann M.C., Morris A.J., Wojciak J.M., Fleming J.K., Karve I., Wright D., Sashindranath M., Goldshmit Y. (2014). Anti-Lysophosphatidic Acid Antibodies Improve Traumatic Brain Injury Outcomes. J. Neuroinflamm..

[221] Ray M., Kihara Y., Bornhop D.J., Chun J. (2021). Lysophosphatidic Acid (LPA)-Antibody (504B3) Engagement Detected by Interferometry Identifies off-Target Binding. Lipids Health Dis..

[222] Goldshmit Y., Matteo R., Sztal T., Ellett F., Frisca F., Moreno K., Crombie D., Lieschke G.J., Currie P.D., Sabbadini R.A. (2012). Blockage of Lysophosphatidic Acid Signaling Improves Spinal Cord Injury Outcomes. Am. J. Pathol..

